# Volatile Metabolome and Transcriptomic Analysis of *Kosakonia cowanii* Ch1 During Competitive Interaction with *Sclerotium rolfsii* Reveals New Biocontrol Insights

**DOI:** 10.3390/microorganisms13071483

**Published:** 2025-06-26

**Authors:** Yoali Fernanda Hernández Gómez, Jacqueline González Espinosa, Griselda Catalina Olvera Rivas, Jackeline Lizzeta Arvizu Gómez, José Humberto Valenzuela Soto, Miguel Angel Ramos López, Aldo Amaro Reyes, Eloy Rodríguez de León, Carlos Saldaña, José Luis Hernández Flores, Juan Campos Guillén

**Affiliations:** 1 Facultad de Química, Universidad Autónoma de Querétaro, Cerro de las Campanas S/N, Querétaro 76010, Mexico; yhernandez01@alumnos.uaq.mx (Y.F.H.G.); jaqui16.je@gmail.com (J.G.E.); golvera13@alumnos.uaq.mx (G.C.O.R.); miguel.angel.ramos@uaq.mx (M.A.R.L.); aldo.amaro@uaq.edu.mx (A.A.R.); eloy.q22@gmail.com (E.R.d.L.); 2 Secretaría de Investigación y Posgrado, Centro Nayarita de Innovación y Transferencia de Tecnología (CENITT), Universidad Autónoma de Nayarit, Tepic 63173, Mexico; jackeline.arvizu@uan.edu.mx; 3 Centro de Investigación en Química Aplicada, Blvd. Enrique Reyna Hermosillo No. 140, Saltillo 25294, Mexico; humberto.valenzuela@ciqa.edu.mx; 4 Facultad de Ciencias Naturales, Universidad Autónoma de Querétaro, Av. De las Ciencias S/N, Querétaro 76220, Mexico; carlos.saldana@uaq.mx; 5 Centro de Investigación y de Estudios Avanzados del IPN, Irapuato 36824, Mexico

**Keywords:** *K. cowanii* Ch1, *S. rolfsii*, RNA-Seq, colonization competence, VOC profile, biocontrol

## Abstract

The volatile organic compounds (VOCs) produced by *K. cowanii* Ch1 play a significant role in the inhibition of the mycelial growth of phytopathogen strains. As a continuation of our previous studies, we aim to elucidate the mechanisms of the responses of *K. cowanii* Ch1 against *S. rolfsii* during a colonization competence interaction in the presence and absence of a mixture of bacterial VOCs under in vitro conditions. The results of this study showed that, in the absence of bacterial VOCs, *K. cowanii* Ch1 cannot compete against *S. rolfsii*, and the RNA-Seq analysis revealed the differential expression of genes related to the oxidative stress response in *K. cowanii* Ch1 for survival. However, in the presence of bacterial VOCs, an interesting phenotypical response was observed in *K. cowanii* Ch1, resulting in the mycelial growth inhibition of *S. rolfsii*. The upregulated genes were related to the siderophore-mediated iron transport system, zinc ion transport system, antibiotic biosynthesis monooxygenase, carbohydrate metabolism, polyketide synthase modules, and related proteins, and *katG* was probably related to the phenotype resulting in the formation of gas bubbles by *K. cowanii*. In addition, the VOC profile analyzed at 36 h for bacterial growth revealed a cocktail with an ability to increase the competence of *K. cowanii* Ch1 against *S. rolfsii* in vitro and in vivo. This study provides evidence regarding the key role that VOCs play during the colonization competition involving *K. cowanii* Ch1, the comprehension of which may enable the development of new biocontrol strategies.

## 1. Introduction

The genus *Kosakonia* is a member of the *Enterobacteriaceae* family [1]. The bacteria of this genus have a metabolic ability to grow as facultative anaerobes, are motile, grow in a wide range of temperatures, ferment various carbohydrates, and have been isolated from diverse ecological niches, which indicates that their metabolic potential in these environments enables competitive colonization against other microorganisms [2] or specific interactions with plant [3], animal [4], fungal [5], or insect hosts [6]. Some common species that reside in the rhizosphere, such as *K. oryzendophytica* YMA7 [7], *K. radicincitans* DSM 16656 [8,9], and *K. oryziphila* NP19 [10], which have been isolated from rice and wheat fields, show important plant growth-promoting traits, such as solubilizing phosphate and potassium, producing IAA and siderophores, and fixing nitrogen. Also, some strains such as *K. cowanii* Ch1 (isolated from chili powder) can produce volatile organic compounds (VOCs); in particular, these VOCs presented important effects on the mycelial growth inhibition of *Alternaria alternata* and *Sclerotium rolfsii*, with a mean rate of 70% under in vitro conditions [11]. Additionally, the *K. cowanii* Cp1 strain isolated from the seeds of *Capsicum pubescens* can produce VOCs during competitive colonization to reduce the soft rot caused by *Pectobacterium aroidearum* SM2 in economically important crops such as chili and tomato fruits [2]. Furthermore, a relevant study has demonstrated that, during the colonization of *K. radicincitans*, the acquisition of nutrients from the plant-beneficial fungus *Serendipita indica* can provide biofilm-based protection against the fungus-feeding bacterium *Collimonas fungivorans* [5]. Research on the colonization of *Anopheles gambiae* and *Glossina* sp. by *K. cowanii* Zambiae from the midgut and its correlation with the production of reactive oxygen intermediates and organic acids has demonstrated its metabolic potential to reduce *Plasmodium* and *Trypanosome* infection in the midgut of these insects [6,12].

Therefore, based on these research findings, a better understanding of the metabolic capabilities of the genus *Kosakonia* has revealed its great potential, not only in interactome systems [13], but also in biotechnological applications to produce important metabolites [14]. In this sense, more research is necessary to advance the knowledge on the bacterial ecological responses occurring during bacterial colonization through metabolomic, transcriptomic, and proteomic analyses under specific growth conditions, such as specific pH, temperature, nutrients, and CO_2_ and O_2_ concentrations, and in environmentally stressful conditions, such as microbial competence, VOC responses, and under toxic metabolites, in order to explore alternative metabolic pathways for the production of novel metabolites, thus providing a competitive advantage that is critical for the survival and colonization of *Kosakonia*.

In this sense, diverse studies have demonstrated the potential of VOCs, produced by an increasing number of microorganisms reported, as potent modulators of communication signals and for the biocontrol of phytopathogenic microorganisms based on their hazardous physical–chemical properties, which may affect cell integrity and the up/downregulation of gene expression related to diverse metabolic pathways, virulence, and alterations in the redox balance that compromise cell viability [15,16]. Therefore, the fact that important chemical classes of VOCs have been identified in *K. cowanii,* such as dodecanoic acid; 3-hydroxy ethanol; 1-butanol-3-methyl; acetaldehyde; butanoic acid, butyl ester; cyclodecane; 2-butanone, 3-hydroxy; disulfide, dimethyl; and pyrazine-2,5-dimethyl, with similar antimicrobial properties and conserved in phylogenetically different bacterial species suggests that there is probably a common competitive colonization strategy that affects the important metabolic functions of phytopathogenic microorganisms, which can eventually be implemented to mitigate the losses caused by microbial infections in crops [17].

Based on our previous study [11], it is imperative to determine whether *K. cowanii* Ch1 requires specific growth conditions or the production of VOCs as modulators of stress responses during competitive interactions with *S. rolfsii*, which is a soil-borne fungus that causes different types of plant diseases, such as stem canker, damping off, crown and root rot, collar rot, foot rot, stem rot, or southern stem rot, in economically valuable crops and produces specialized structures called sclerotia, which enhance its spread in the field during the disease cycle and represent a significant challenge for disease control associated with this microorganism [18]. Therefore, these findings will provide new insights into the biocontrol of *S. rolfsii* or other fungal strain such as *A. alternata* in the field as an alternative of toxic chemical fungicides [17].

Therefore, the aim of this study was to determine whether (i) *K. cowanii* Ch1 can compete directly with *S. rolfsii*, (ii) if the presence of bacterial VOCs during bacteria–fungal interaction produce a beneficial competitive interaction to *K. cowanii* Ch1, and (iii) to determinate gene expression changes in *K. cowanii* Ch1 during microbial interaction. To achieve this aim, we conducted in vitro bacterial–fungal interaction assays to evaluate whether the presence of bacterial VOCs can enhance the efficiency of the colonization competence of *K. cowanii* Ch 1 to reduce mycelial growth. Additionally, we investigated the transcriptional profiling of *K. cowanii* Ch1 using RNA-Seq during these bacterial–fungal interactions for the elucidation of competence responses.

## 2. Materials and Methods

### 2.1. Bacterial–Fungal Interaction Assays

The fungal strains evaluated were *Sclerotium rolfsii* and *Alternaria alternata,* both of which present mycelial growth inhibition in response to the VOCs produced by *K. cowanii* Ch1, as well as *Fusarium oxysporum*, which presents VOC resistance in previous work [11]. These fungal strains were provided and identified by their morphological characteristics by the Laboratory of Plants and Agriculture Bio-technology at Queretaro University, Mexico. The fungal strains were grown in a potato dextrose agar (PDA) medium (Difco Laboratories, Detroit, MI, USA) and incubated at 28° for 5–7 days as per the requirements of each specific fungal strain. The bacterial strain used was *K. cowanii* Ch1 [11], and the bacterial strains used as controls for VOC production were *Bacillus altitudinis* CH05 [19], *Bacillus tropicus* CH13 [19], and *Pectobacterium aroidearum* SM2 [2], which were grown in a TSA medium (Difco Laboratories, Detroit, MI, USA) at 37 °C. For bacterial–fungal interaction assays, a double-compartment Petri dish chamber (9 cm) was used [11]. For the treatments, the lower part of the compartment was inoculated with 100 µL of each bacterial strain (1 × 10^8^ CFU/mL) in the TSA medium (Difco Laboratories, Detroit, MI, USA) for VOC production, while in the upper compartment, the PDA medium (Difco Laboratories, Detroit, MI, USA) was inoculated with fungal strain disks (with a diameter of 7 mm). Around these fungal disks, 20 µL (1 × 10^8^ CFU/mL) of *K. cowanii* Ch1 was inoculated. For the treatments without VOC production, the PDA medium (Difco Laboratories, Detroit, MI, USA) was inoculated with fungal strain disks, and 20 µL (1 × 10^8^ CFU/mL) of *K. cowanii* Ch1 was inoculated around each of these fungal disks, while the lower compartment was not inoculated with any bacterial strains, such that VOC production was absent in that compartment. For the controls, each fungal disk and *K. cowanii* Ch1 (in the upper compartment) were grown with or without the production of bacterial VOCs (in the lower compartment). All double-compartment Petri dish chambers were sealed with parafilm and incubated at 28 °C, and measurements of the radial mycelial growth were performed every 24 h.

### 2.2. Analysis of VOCs Produced by Bacterial Strains via HS-SPME-GC-MS

The VOC profiles produced by the bacterial strains were evaluated at 24 h of growth in 50 mL of tryptone soy broth (TSB) medium at 28 °C and 120 rpm with an initial inoculum of 1 × 10^4^ CFU/mL. The characterization of VOCs was performed using a previously reported methodology [11], where the samples were incubated at 50 °C for one hour and then the VOCs were collected on a divinylbenzene/carboxen/polydimethylsiloxane fiber (DVB/CAR/PDMS, Supelco, Sigma-Aldrich, Visalia, CA, USA). After this, manual injection was carried out in splitless mode; the injection port and transfer line temperature were set to 250 °C using a 7820A GC with a 5975C MSD (Agilent Technologies, Inc., Santa Clara, CA, USA) and HP-5MS 30 m, 0.25 mm, and 0.25 µm GC Column Capillaries (Agilent Technologies Inc., Santa Clara, CA, USA). The column oven was programmed at 40 °C, increasing to 180 °C at 5 °C/min and then at 20 °C/min to 260 °C, and held at that temperature for 5 min. Helium (99.999% purity) was used as the carrier gas with a flow rate of 1.0 mL/min. Mass spectrometry analyses were conducted at an electron energy of 70 eV and the *m*/*z* range was 33–500. Data were obtained and processed using the NIST/EPA/NIH Mass Spectra Library instrumental analysis software, version 2017, Antioch, CA, USA. For comparison of the VOCs between bacterial strains, the compounds with the highest relative abundance values (%) were plotted with the statistical R program (version 4.2.2) using ggplot2 and pheatmap. Based on the VOC profiles detected using the described methodology, we tested the following synthetic VOCs to evaluate their phenotypical responses in *K. cowanii* Ch1 during its physical interaction with *S. rolfsii*. A Petri dish of 9 cm in diameter was used to place a sterile filter paper disk containing the synthetic VOCs, while another Petri dish with potato dextrose agar (PDA) medium (Difco Laboratories, Detroit, MI, USA) was co-inoculated with the bacteria-fungal strain. The volume of each VOC used was as follows: acetoin of 100 µL (≥95%, Sigma-Aldrich, USA), 2,5-Dimethyl pyrazine of 50 µL (98%, Sigma-Aldrich, USA), ethanol of 200 µL (≥99.5%, Sigma-Aldrich, USA), cyclododecane of 50 µL (a solution prepared with 20 mg cyclododecane dissolved in 1 mL of hexane), and benzaldehyde of 20 µL (97%, Sigma-Aldrich, USA).

### 2.3. Evaluation of Cell-Free Filtrates in In Vitro and In Vivo Competitive Colonization Interaction Essays

*K. cowanii* Ch1 was grown for 48 h on the tryptic soy broth medium (Difco Laboratories; Detroit, MI, USA) at 28 °C and 100 rpm for 48 h. Cell-free filtrates were obtained at 12, 24, 36, and 48 h of growth via centrifugation and filtration with 0.22 μm pore-size disposable filters (Corning Incorporated, Corning, NY, USA). To evaluate the cell-free filtrates in vitro, first, the fungal mycelium disk and *K. cowanii* Ch1 inoculated around it were grown in the potato dextrose agar (PDA) medium (Difco Laboratories, Detroit, MI, USA) for 24 h at 28 °C. After this period of incubation, 50 µL of each cell-free filtrate was added around the mycelium disks every 12 h for 2 days, and the results were recorded on the 5th day. As a control, a fungal mycelium disk was grown in the PDA medium (Difco Laboratories, Detroit, MI, USA). From these experiments, the cell-free filtrate at 36 h showed the highest mycelium growth inhibition and was used for in vivo experiments. The VOC profile produced at 36 h was analyzed according to the methodology described in Section 2.2 and compared with the VOC profile obtained at 24 h for *K. cowanii* Ch1. To evaluate the cell-free filtrates in vivo, bacterial–fungal competitive colonization interaction assays were performed on fruits of the serrano chili (*Capsicum annuum* L.) to assess the potential of *K. cowanii* Ch1 against *S. rolfsii* in reducing infection symptoms. The serrano chili fruits were surface sterilized as previously reported [2], then punctured with a sterile toothpick and inoculated with 10 µL of *K. cowanii* Ch1 suspension at a concentration of 1 × 10^8^ CFU/mL; this assay was performed in triplicate. After bacterial inoculation, three mature melanized sclerotia were inoculated at the same point. The controls were inoculated only with mature melanized sclerotia. To determine whether cell-free filtrates possessed activity against fungal growth and reduce infection, the chili fruits were inoculated with sclerotia or co-inoculated with sclerotia–bacteria and incubated at 28 °C for 24 h. After this period of incubation, a 20 µL volume of cell-free filtrates was applied during bacterial–fungal treatments of infections at 12, 24, and 36 h. The inoculated chili fruits were placed inside an airtight container at 28 °C. The infection of *S. rolfsii* in chili fruits was observed as macerated tissue and the diameter was registered in millimeters for comparison with the control and treatments.

### 2.4. RNA Isolation

Based on the results of microbial interaction obtained, we decided to use the bacterial cells at 36 h of interaction, because we could recover the bacterial colony for the RNA-Seq analysis; beyond 36 h, a thick mycelium was established in the absence of bacterial VOCs, which made bacterial recovery difficult in comparison. The bacterial cells representing technical duplicates of treatments in (1) the absence of bacterial VOCs, and (2) the presence of bacterial VOCs and control (*K. cowanii* Ch1 growth in the absence of VOCs) were scraped into 800 µL of RNA Shield^TM^ reagent (Zymo Research, Irvine, CA, USA), resuspended, and briefly vortexed, before being stored at 5 °C. The total RNA was purified using the Quick-RNA™ Miniprep Plus Kit, following the manufacturer’s instructions (Zymo Research, Irvine, CA, USA).

### 2.5. RNA-Seq Library Preparation

RNA samples were sent to Zymo Research, Irvine, CA, USA, for total RNA-Seq service. Libraries were constructed from the total RNA samples. Libraries were prepared using the Zymo-Seq RiboFree Total RNA Library Prep Kit^TM^, according to the manufacturer’s instruction manual. Briefly, RNA was reverse transcribed into cDNA, which was followed by ribosomal RNA depletion. After that, a partial P7 adapter sequence was ligated at the 3′ end of cDNAs, followed by second-strand synthesis and partial P5 adapter ligation to the 5′ end of the double-stranded DNA. Lastly, libraries were amplified to incorporate full-length adapters under the following conditions: initial denaturation at 95 °C for 10 min; 10–16 cycles of denaturation at 95 °C for 30 s, annealing at 60 °C for 30 s, and extension at 72 °C for 60 s; and final extension at 72 °C for 7 min. Successful library construction was confirmed with Agilent’s D1000 ScreenTape Assay on TapeStationc (Agilent Technologies, Inc., Santa Clara, CA, USA). The RNA-Seq libraries were sequenced on an Illumina NovaSeq to a sequencing depth of at least 30 million read pairs (150 bp paired-end sequencing) per sample.

### 2.6. RNA-Seq Data Bioinformatics Analysis

RNA-Seq data were analyzed at Zymo Research using the RNA-Seq pipeline adapted from the nf-core/rnaseq pipeline v1.4.2 (https://github.com/nf-core/rnaseq, accessed on 11 November 2024). The pipelines were built using Nextflow [20]. Briefly, the quality control of raw reads was carried out using FastQC v0.11.9 [21]. Adapter and low-quality sequences were trimmed from raw reads using Trim Galore! v0.6.6 [21]. Trimmed reads were aligned to the reference genome of *K. cowanii* Ch1 (genome accession number at NCBI: JAUDFU000000000) using STAR v2.6.1d [22]. BAM file filtering and indexing were carried out using SAMtools v1.9 [23]. RNAseq library quality control was implemented using RSeQC v4.0.0 and QualiMap v2.2.2-dev [24,25]. Duplicate reads were marked using Picard tools v2.23.9 [26]. Library complexity was estimated using Preseq v2.0.3 [27]. Duplication rate quality control was performed using dupRadar v1.18.0 [28,29]. Differential gene expression analysis was completed using DESeq2 v1.28.0 with a padJ of ≤0.05, where the mean transformed read counts of genes, to normalize sequencing depth and RNA composition, was used in DESeq2. The similarities (Pearson correlation coefficient) between samples were calculated using the normalized and ‘rlog’ transformed read counts of all genes via DESeq2. Also, multidimensional scaling was conducted to visualize the distance/similarity between samples [30]. Quality control and analysis result plots were visualized using MultiQC v1.9 [31]. Functional enrichment analysis was achieved with ShinyGO 0.80 [32], in which genes with a fold change of >1.0 and an FDR of <0.05 present in the pathway database in the Local Network Cluster (STRING) were considered as highly differentially expressed. The raw data has been deposited in the NCBI SRA database with the BioProject accession number PRJNA1276248.

### 2.7. Statistical Analysis

At least three technical replicates of in vitro and in vivo competitive colonization interaction assays were carried out for statistical analysis. The equation ICM = [(C) − (T)/C] × 100% was used to analyze the growth percentage of the controls (C) and treatments in the presence or absence of VOCs in vitro or in vivo experiments to determine the infection percentage (T), and the data were analyzed using Minitab version 18.0. Means with ±standard error were analyzed via one-way ANOVA (*p* < 0.05).

## 3. Results

### 3.1. Bacterial–Fungal Interaction Assays

**Figure 1:**
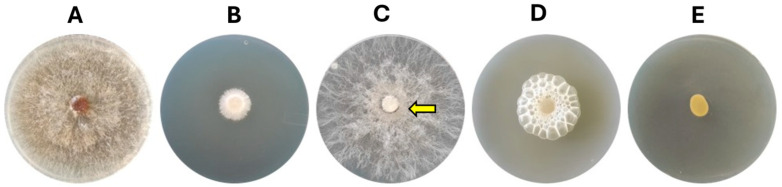
Microbial interaction assays. (**A**) *S. rolfsii* was grown in a PDA medium. (**B**) *S. rolfsii* was grown in the presence of bacterial VOCs, where mycelial growth was affected. (**C**) *K. cowanii* Ch1 grew around the mycelial disk in the absence of bacterial VOCs and (**D**) *K. cowanii* Ch1 grew around the mycelial disk in the presence of bacterial VOCs. The arrow in (**C**) indicates that *S. rolfsii* has outgrown the bacterial colony in the absence of VOCs, while in the presence of VOCs (**D**), gas bubbles were produced by *K. cowanii* Ch1. (**E**) As a control, the bacterial colony was grown in the presence of VOCs. The images were taken at 72 h.

*K. cowanii* Ch1 can produce VOCs with antifungal activity, and its effects were evaluated on the mycelial growth in *S. rolfsii*. The result showed an inhibition with a mean rate of 80 ± 5% (*p* < 0.05) at 72 h when compared with the control (Figure 1A,B). With this result in mind, we decided to test whether *K. cowanii* Ch1 competed against *S. rolfsii* during a physical interaction and reduced the mycelial growth; thus, we conducted an assay where *K. cowanii* Ch1 was grown around a mycelial disk of *S. rolfsii*. As indicated in Figure 1, contrary to the first experiment conducted, *K. cowanii* Ch1 did not affect the mycelial growth and *S. rolfsii* outgrew the bacterial colony (Figure 1C, indicated by an arrow). Therefore, we decided to test whether, for an attenuated mycelial growth disturbed probably by oxidative stress caused by bacterial VOCs, *K. cowanii* Ch1 had a major effect in reducing the mycelial growth during physical interactions. Surprisingly, the metabolic response of *K. cowanii* Ch1, through the growth around the mycelial fungal disk, was evident, with gas bubble production being observed; indeed, the complete mycelial growth inhibition was observed, as indicated in Figure 1D. This phenotypical trait was not observed in the bacterial colony in the presence of VOCs (Figure 1E).

**Figure 2:**
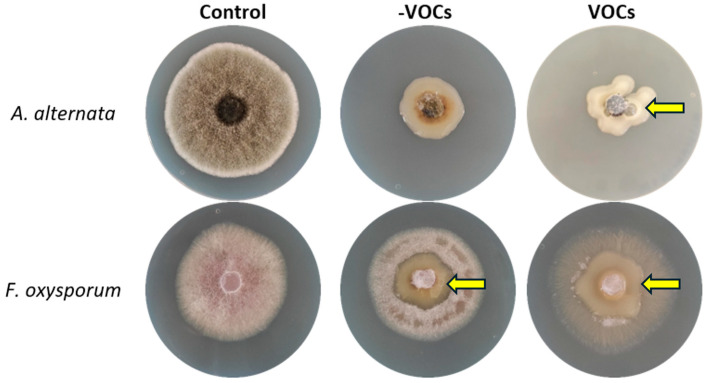
Evaluation of *K. cowanii* Ch1 against *A. alternata* and *F. oxysporum*. Fungal strains were grown under the presence or absence of VOCs produced by *K. cowanii* Ch1. In both conditions, *K. cowanii* Ch1 was grown around the mycelial disks. The arrow shows the gas bubbles produced with *A. alternata*. However, for *F. oxysporum*, the arrows indicate that the mycelium outgrew the bacterial colony. Controls indicate the fungal strains’ growth without VOCs.

Based on these results, we decided to evaluate whether the additional fungal strains would have similar metabolic responses, or whether they were exclusively produced by *S. rolfsii*. Thus, we tested *A. alternata*, a fungal strain also sensitive to VOCs produced by *K. cowanii* Ch1, and it showed a mean rate of mycelial growth inhibition of 70 ± 5% (*p* < 0.05) compared with that of the control. A similar metabolic response was observed in the presence of VOCs with the production of gas bubbles by *K. cowanii* Ch1 (Figure 2, indicated by an arrow), but not in the absence of VOCs. Both conditions showed mycelial growth inhibition as indicated in Figure 2. On the other hand, *F. oxysporum*, a fungal strain with resistance to VOCs produced by *K. cowanii* Ch1, showed a mean rate of mycelial growth inhibition of 10 ± 5% (*p* < 0.05) compared with that of the control; the production of gas bubbles by *K. cowanii* Ch1 was not observed in the presence of VOCs, and the mycelium outgrew the bacterial colony without affectation (Figure 2, indicated by an arrow). This result indicated that *F. oxysporum* possessed different mechanisms of resistance to VOCs produced by *K. cowanii* Ch1, and that sensitive fungal strains probably produce important molecules in response to oxidative stress, which could compromise their cell viability.

**Figure 3:**
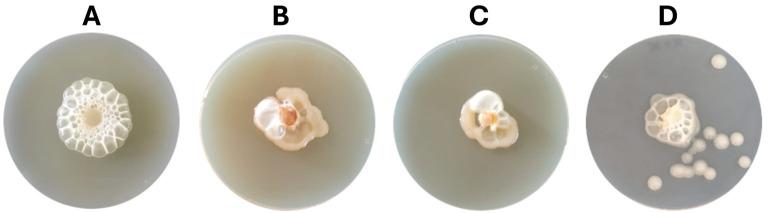
Evaluation of VOCs produced by bacterial strains. VOCs produced by (**A**) *K. cowanii* Ch1, (**B**) *B. altitudinis* CH05, (**C**) *B. tropicus* CH13, and (**D**) *P. aroidearum* SM2. In all conditions, *K. cowanii* Ch1 was grown around mycelial disks of *S. rolfsii*.

With these evident results—and the fact that sensitive fungal strains had similar oxidative stress responses under these bacterial VOCs—we decided to test whether *K. cowanii* Ch1 produced specific VOCs compared with other bacterial strains that produced some common VOCs with a similar effect on the mycelial growth inhibition of *S. rolfsii*. Therefore, we evaluated the VOCs produced by *B. altitudinis* CH05, *B. tropicus* CH13, and the phytopathogen *P. aroidearum* SM2. The results shown in Figure 3 revealed similar results in *S. rolfsii* under this mixture of VOCs produced in all bacterial strains, as well as the phenotypical response of gas bubble production in *K. cowanii* Ch1 when it grew around the mycelial disks. However, an increased response was observed with the VOCs produced by *K. cowanii* Ch1 (Figure 3A) and *P. aroidearum* SM2 (Figure 3D). Additionally, complete mycelial growth inhibition was observed under these conditions.

### 3.2. Identification of VOCs Using HS-SPME-GC-MS

**Figure 4:**
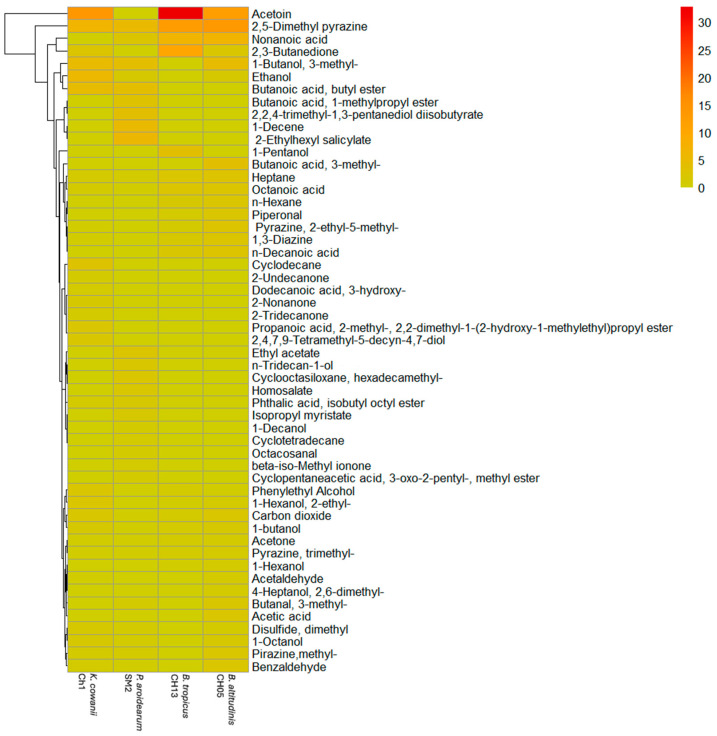
Relative percentages of the different classes of VOCs produced in the bacterial strains at 24 h. The bar color represents the relative abundance based on the relative peak area (%) detected in the HS-SPME-GC-MS analysis.

We compared the VOCs produced at 24 h by the bacterial strains to identify some common molecules (Figure 4 and Table S1). A total of 53 compounds were detected and plotted in Figure 4, which were classified as alcohols (18.10%), aldehydes (7.56%), acids (16.50%), pyrazines (15.28%), ketones (7.87%), hydrocarbons (7.44%), thiols (1.13%), and other compounds (24.75%). The molecules with a high relative abundance in *B. tropicus* CH13 were acetoin (32.77%), 2,5-Dimethyl pyrazine (12.33%), 2,3-Butanedione (10.38%), and Nonanoic acid (6.53%); in *B. altitudinis* CH05 were 2,5-Dimethyl pyrazine (13.31%), Acetoin (11.9%), Nonanoic acid (6.72%), and 3-methyl-1-Butanol (4.43%); in *P. aroidearum* SM2 were 2-Ethylhexyl salicylate (5.65%), 2,5-Dimethyl pyrazine (4.99%), 1-Decene (4.93%), and Butanoic acid, butyl ester (4.25%); and in *K. cowanii* Ch1 were acetoin (13.52%), 2,5-Dimethyl pyrazine (6.47%), Ethanol (5.56%), and 3-methyl-1-Butanol (4.85%). Based on the profiles of the VOCs and whether some of them can cause oxidative stress in *S. rolfsii*, we decided to use acetoin, 2,5-Dimethyl pyrazine, ethanol, cyclododecane, and benzaldehyde to test the bacterial–fungal interactions as above, but the phenotypical response in *K. cowanii* Ch1 was not observed; thus, the complex mixture of VOCs probably had multiple targets in the cells to cause oxidative stress (Figure S1).

### 3.3. In Vitro and In Vivo Evaluation of Cell-Free Filtrates

**Figure 5:**
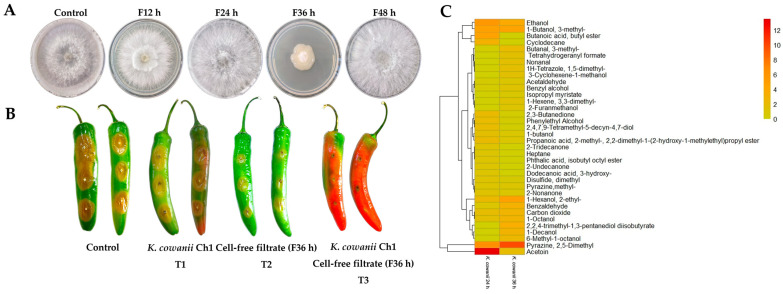
The in vitro and in vivo evaluation of cell-free filtrates. Cell-free filtrates obtained at 12, 24, 36, and 48 h of bacterial growth were added during fungi–bacteria interaction (**A**). (**B**) Colonization competence between both microorganisms was evaluated in chili fruits (T1). Cell-filtrate obtained at 36 h was evaluated against *S. rolfsii* (T2) and T3 is the co-inoculation of both microorganisms treated with the cell-free filtrates (F36 h). Control was inoculated only with *S. rolfsii*. Results in (**A**) were recorded on the 5th day of mycelial growth, and results in (**B**) were recorded on the 4th day of the experiment. (**C**) A comparison of VOC profiles produced at 24 h and 36 h in *K. cowanii* Ch1. The bar color represents the relative abundance based on the relative peak area (%) detected in the HS-SPME-GC-MS analysis.

Based on the microbial interaction in the presence of VOCs (Figure 1), we decided to evaluate cell-free filtrates from *K. cowanii* Ch1 obtained during the different bacterial growth time periods and applied directly on the physical interaction between the mycelium disk and the bacteria growth on the PDA medium to determine its response to the inhibition of mycelium growth in vitro (Figure 5A). We observed that the cell-free filtrate obtained at 36 h of bacterial growth showed an important mycelial inhibition capacity compared with the control and the other cell-free filtrates (12, 24 and 48 h) recorded on the 5th day of the experiment. Thus, we decided to evaluate this cell-free filtrate (36 h) on the bacterial–fungal competitive colonization interaction on fruits of the serrano chili (*Capsicum annuum* L.) to determine the potential of *K. cowanii* Ch1 to reduce infection symptoms caused by *S. rolfsii* (Figure 5B). When *K. cowanii* Ch1 was co-inoculated with *S. rolfsii* (T1), an evident infection symptom was observed with a diameter of 1.8 ± 0.2 cm, similar to the control with a diameter of 2.2 ± 0.3 cm. In treatment T2, the cell-free filtrate (F36 h) showed a reduction in infection symptoms of nearly 50% (1.06 ± 0.8 cm). When both microorganisms were co-inoculated and treated with a cell-free filtrate (T3), a reduction of 80% of infection symptoms was observed (0.5 ± 0.2 cm) compared with the control. These results mean that biomolecules produced around 36 h of bacterial growth can inhibit mycelial growth, but in the presence of bacteria, additional metabolic activity during competitive colonization can increase major fungal inhibition.

To adequately understand the differences between cell-free filtrates, we decided to analyze the VOC profile at 36 h of bacterial growth. The comparative results of the VOCs produced at 36 h and 24 h of bacterial growth showed important differences in the VOC chemical classes (Figure 5C and Table S2). For example, Butanoic acid, butyl ester; 2-Undecanone; Cyclodecane; 2-Tridecanone; Dodecanoic acid, 3-hydroxy-; Heptane; and Phthalic acid, isobutyl octyl ester were absent at 36 h. Other chemicals, such as Ethanol; 2,3-Butanedione; 1-butanol; acetoin; Phenylethyl Alcohol; Disulfide, dimethyl; 2-Nonanone; Propanoic acid, 2-methyl-, 2,2-dimethyl-1-(2-hydroxy-1-methylethyl)propyl ester; and 2,4,7,9-Tetramethyl-5-decyn-4,7-diol were reduced considerably at 36 h. The chemical compounds that increased or were synthetized de novo with a high relative abundance in percentage were Pyrazine, 2,5-Dimethyl; 1-Decanol; 2,2,4-trimethyl-1,3-pentanediol diisobutyrate; Isopropyl myristate; Acetaldehyde; Butanal, 3-methyl-; Benzaldehyde; 1-Octanol; 1-Hexanol, 2-ethyl-; Nonanal; 1-Hexene, 3,3-dimethyl-; 6-Methyl-1-octanol; Tetrahydrogeranyl formate; 1H-Tetrazole, 1,5-dimethyl-; 2-Furanmethanol; 3-Cyclohexene-1-methanol; and Benzyl alcohol. Therefore, the observed differences in the composition and concentrations of the VOCs at different time periods of bacterial growth are important research findings, as VOCs could have a major impact on microbial interactions and colonization competence.

### 3.4. RNA Sequencing Analysis in K. cowanii Ch1

**Figure 6:**
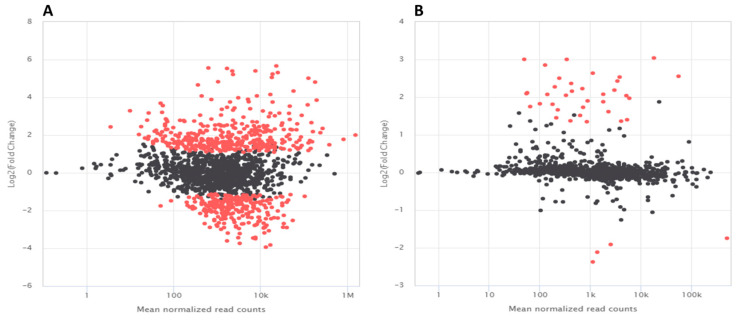
The MA plot visualization of differential gene expression in *K. cowanii* Ch1. Differentially expressed genes in pairwise comparisons during bacterial–fungal interactions in the absence of VOCs (**A**) and in the presence of VOCs (**B**). These conditions were compared with *K. cowanii* Ch1 grown in the absence of VOCs as a control. Expression levels are shown on X-axis, while log2 of fold changes are shown on Y-axis. Red dots represent differentially expressed genes (adjusted *p*-values of <0.05). Black dots represent non-differentially expressed genes. The shrinkage of effect size was carried out using the ‘ashr’ method in DESeq2.

According to the results obtained in Figure 1C,D, and in order to gain insight into the competence mechanism of *K. cowanii* Ch1 against *S. rolfsii*, the bacterial–fungal interaction assays in the presence or absence of VOCs were performed to study the bacterial transcriptome at 36 h of the interaction. By this time, the initial mycelium had outgrown the bacterial colony in the absence of VOCs, and we could recover the bacterial colony for the RNA-Seq analysis; beyond 36 h a thick mycelium was established, which made bacterial recovery difficult. Approximately 47.50 million paired-end sequence reads were generated from each duplicate technical condition and the percentage of reads successfully mapped against the reference genome were from 24.3% to 98.9% (Table S3). The similarities between samples are visualized in the form of a heatmap in Supplementary Figure S2 and the mean transformed read counts of genes is shown in Supplementary Figure S3. The general statistics of differentially expressed genes in pairwise comparisons (adjusted *p*-values of <0.05) showed that, in the absence of VOCs, 388 genes were upregulated, and 269 genes were downregulated (Figure 6A), with 3796 genes that were not differentially expressed. However, in the presence of VOCs, 35 genes were upregulated, and 4 genes were downregulated (Figure 6B), with 4414 genes that were not differentially expressed.

### 3.5. Differentially Expressed Genes in K. cowanii Ch1 in Absence of VOCs

**Figure 7:**
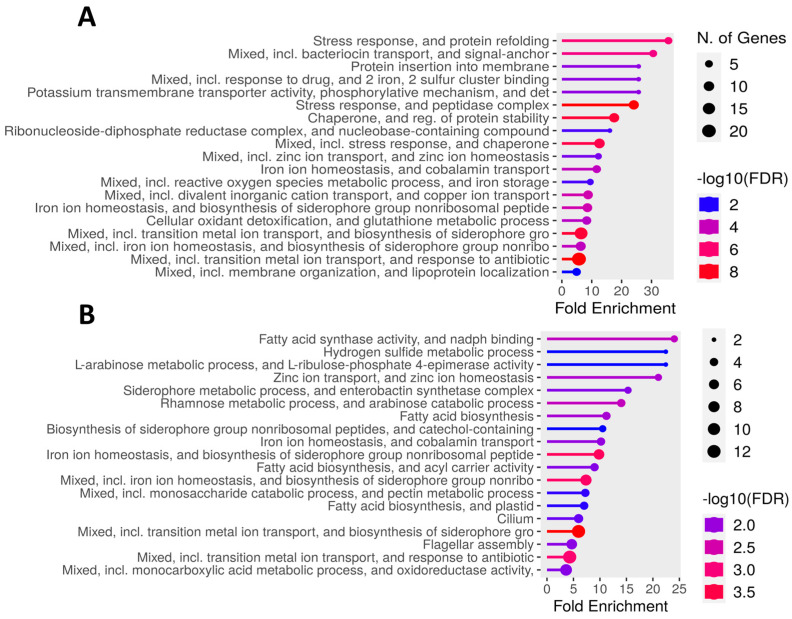
Gene ontology (GO) enrichment. Pathways detected for microbial interaction in the absence of VOCs (**A**) and in the presence of VOCs (**B**). Genes with a fold change of >1.0, an FDR of <0.05, and present in the pathway database, the Local Network Cluster (STRING), were considered as highly differentially expressed.

According to the differentially upregulated genes in *K. cowanii* Ch1, gene ontology (GO) enrichment analysis was carried out, and the top enriched pathways were listed in Figure 7A. During this microbial competence interaction, 19 top pathways were identified, and diverse genes were related to the stress responses, which indicated that *K. cowanii* Ch1 was under very stressful conditions when interacting with the fungal strain. Important key transcriptional regulators were detected, such as the yqjI gene (with a more than four-fold change), which is related to iron homeostasis. Furthermore, the feoC gene was upregulated by two-fold, which is related to the mediation of ferrous iron [Fe(II)] import. The redox-sensitive transcriptional regulators soxRS and qorR were upregulated, with a >1.5-fold change. The phoP gene was upregulated by two-fold, which is related to virulence, Mg^2+^ homeostasis, and resistance to a variety of antimicrobial agents, including acidic pH and cationic antimicrobial peptides. The zntR gene was upregulated by two-fold, which is related to the control of cellular Zn(II) status. The ydcI gene was upregulated by two-fold, which is a LysR family transcriptional regulator related to pH homeostasis. The anaerobic nitric oxide reductase transcription regulator norR gene was upregulated by two-fold, which encodes a σ^54^-dependent regulatory protein that senses nitric oxide to activate detoxification genes. The response regulator creB gene was upregulated by nearly two-fold, which is part of a two-component signal transduction system CreBC (for carbon source responses), which is a global sensing and regulation system that controls the expression of genes involved in a variety of functions, including the enzymes of intermediary catabolism. The LysR family transcriptional regulator yeiE was upregulated, which is related to the regulation of flagellum-mediated motility. The transcriptional regulator slyA was upregulated, which is involved in the regulation of genes that are important for bacterial virulence and stress response. The iron–sulfur cluster regulator iscR gene is a sensor of cellular [Fe-S] levels and a global transcription regulator for [Fe-S] homeostasis under stressful conditions.

Following these routes of the oxidative stress defense system and the activation of diverse genes by these key transcriptional regulators, diverse pathways were observed (Figure 7A). Some of them were related to the siderophore-mediated iron transport, and these genes were upregulated with a >1.5-fold change, such as entCF that encodes synthetases for the biosynthesis of enterobactin. Enterobactin recognition and transport are represented by upregulated genes such as fepBCD, tonB, exbBD, and fes. Additionally, the ferrous iron transport system was represented by upregulated genes such as feoAB. Genes that encode for oxidative enzymes were upregulated with a >1.5-fold change and included 2-Oxobutyrate oxidase (ID QU629_RS16930), Ferric reductase (fhuF), Alkyl hydroperoxide reductase protein C (ahpC), NAD-dependent glyceraldehyde-3-phosphate dehydrogenase (gapA), Peptide-methionine (S)-S-oxide reductase (msrA), Ribonucleotide reductase of class III (nrdG), Thiol peroxidase (tpx), Oxidoreductase (ID QU629_RS03245), and other important genes (Table S2). The genes upregulated with a >1.5-fold change that prevented protein misfolding and aggregation were represented by the multiple stress resistance protein BhsA (bhsA), 16 kDa heat shock protein AB (ibpAB), heat shock protein GrpE (grpE), chaperone protein ClpB (ATP-dependent unfoldase) (clpB), chaperone protein DnaJK (dnaJK), heat shock protein 60 kDa family chaperone GroEL (groL), and HtrA protease/chaperone protein (degP). The cellular oxidant detoxification and glutathione metabolic process pathways were represented by Glutathione S-transferase (gstA), Thioredoxin 2 (trxC), and Glutaredoxin-like protein NrdH (nrdH). Endonuclease IV was upregulated with a 5-fold change. Downregulated genes related to important transporters, membrane proteins, motility, signal regulation, and metabolism were detected (Table S4).

### 3.6. Differentially Expressed Genes in K. cowanii Ch1 in Presence of VOCs

Based on the evident phenotype shown by *K. cowanii* Ch1 under the presence of VOCs (Figure 1D), the RNA-Seq analysis revealed that, during its microbial competence interaction, 19 top pathways were identified (Figure 7B, Table S5). Also, and probably related to the phenotype of bubble gas of *K. cowanii* Ch1, the gene katG that encodes for a catalase is upregulated by 2.7-fold. This means, under this specific competence interaction, a probable hypothesis is that fungal strains may produce hydrogen peroxide (H_2_O_2_) as a VOC stress response. Therefore, the antioxidant systems that remove H_2_O_2_ are activated in *K. cowanii* Ch1. In addition, stress response genes were identified in the metal transportation mechanisms, with a >1.0-fold change in gene upregulation, siderophore-mediated iron transport system (entS, entCF, fepBDG, tonB, exbBD and fes) and ferrous iron transport system (feoAB, efeOU). The zinc ion transport system and homeostasis were represented by genes upregulated with a >1.0-fold change, such as znuABC. Protein misfolding and aggregation were represented by the 16 kDa heat shock protein AB (ibpAB). The flagellar assembly genes detected were flhACD, flgCDEFGHI, and filFN. Genes related to the production of biomolecules with antimicrobial responses were detected, such as antibiotic biosynthesis monooxygenase (ID QU629_RS21570), which was upregulated with a 2.1-fold change; furthermore, polyketide synthase modules and related proteins (ID QU629_RS05045) were upregulated with a 1.7-fold change. Additional upregulated and downregulated genes related to membrane proteins, signal regulation, and metabolism were detected (Table S5).

## 4. Discussion

Understanding the beneficial bacterial responses during the colonization competition against phytopathogenic microorganisms could be crucial not only for elucidating genetic mechanisms during microbial interactions, but also for developing effective strategies that could enhance the metabolic abilities and eventually be applied to reduce disease symptoms in crops in agricultural systems, as an alternative to the use of undesirable chemical fungicides [17]. The increased research interest for identifying colonization factors (CFs) in specific ecological niches has allowed for the development of diverse methodology approaches [33]; however, significant challenges remain to be overcome. Therefore, in this study, we hypothesized that the VOCs produced by *K. cowanii* Ch1 could be modulators of stress responses during the competitive colonization interaction against *S. rolfsii*. In this sense, we developed microbial interaction assays to overcome the challenges, and the results indicated potential key biological processes that may contribute to a better understanding of biocontrol.

Based on the microbial interaction assays and according to our results shown in Figure 1B, a mixture of VOCs represented by a diverse chemical class were identified (Figure 4), which were produced by *K. cowanii* Ch1 to arrest mycelial growth at a mean rate of 80 ± 5% (*p* < 0.05) in *S. rolfsii*. Thus, based on this bacterial trait, a possible alternative to biocontrol, for preventing fungal disease caused by *S. rolfsii,* could be devised. However, in agreement with the diverse research conducted in this field, the assays of inhibition of mycelial growth by the bacterial VOCs produced in vitro could be the first step to selecting potential microorganisms under controlled conditions; additional research is required to be conducted, as the experimental conditions may not be representative of real growth conditions. Diverse studies have also confirmed that environmental conditions, including pH, CO_2_/O_2_ levels, nutrients, temperature, humidity, growth stage, and interactions between microorganisms of different species, can influence the composition of these mixtures of volatiles produced by the studied microorganisms [34,35,36,37]. In this sense, our results clearly showed that, under the tested conditions, the profile of volatile compounds differed between bacterial species, and even closely related *Bacillus* strains produced different mixtures of volatile compounds. Some common molecules with different concentrations were analyzed at least at 24 h (Figure 4 and Table S1), and they had the ability to impact the inhibition of mycelial growth during the interaction between *K. cowanii* Ch1 and *S. rolfsii* (Figure 3). Also, both fungal strains *S. rolfsii* and *A. alternata* were sensitive to the VOCs produced by some of the tested bacterial strain as previously reported [11,19], while *F. oxysporum* showed resistance to these mixtures of volatiles, demonstrating different evolutionary mechanisms to counteract compounds with hazardous physical–chemical properties that may affect cell integrity and viability. Therefore, future perspectives on molecular processes and the comparison of mechanisms between sensitive and resistant fungal strains could provide us with possible new routes to design better strategies against these resistant strains.

Colonization competence evaluated between *K. cowanii* Ch1 and *S. rolfsii* (Figure 1C) in the absence of VOCs revealed an important finding for understanding why the *K. cowanii* Ch1 colony was outgrown by the mycelium of *S. rolfsii*. Under this microbial interaction condition, gene expression analysis showed that complex defense and repair mechanisms were induced by *K. cowanii* Ch1 to mitigate the oxidative stress induced by the mycelial growth (Figure 7A, Table S4). In this regard, the redox-sensitive transcriptional regulator SoxR (*soxR*) was induced by 2.2-fold, and its critical role in the defense system, under oxidative stress related to enteric bacteria, has been well studied [38,39]. When SoxR is activated through the oxidation of [2Fe-2S] clusters, it induces the expression of the *soxS* gene [39], a DNA-binding transcriptional dual regulator which, in our results, was induced by 1.5-fold, and has the ability to activate the transcription of diverse genes related to the restoration of redox homeostasis and the repair of cellular damage induced by oxidative stress [38,39]. Protective mechanisms observed in *K. cowanii* Ch1 induced a 5.8-fold change in Endonuclease IV (QU629_RS04135), a DNA damage-specific endonuclease that is induced by oxidative stress in related enteric bacteria [40]. Molecular chaperones (*ibpAB*, *grpE*, *clpB*, *dnaJK*, *groL*, *degP*) were also upregulated, and they play a regulatory role in the folding of proteins, the intracellular transport of proteins, the repair and degradation of proteins, and the refolding of proteins during specific cell growth conditions, but were markedly upregulated by stress effectors [41]. The multiple stress resistance gene *bhsA* was induced by five-fold, which is a putative outer membrane protein that has been studied in *E. coli* and shown to influence biofilm formation through hydrophobicity and stress response [42]. The mechanisms of metal homeostasis were probably induced as metals are important antimicrobial effectors that resist cellular stress for survival; for example ZnuABC system was upregulated by five-fold, which indicates that ZnuABC is essential for zinc uptake and required as cofactor in catalytic sites of enzymes related with oxidative stress [43,44]. Additionally, the iron uptake systems (*fepBCD*, *tonB*, *exbBD*, *fes* and *feoAB*) and genes related to the production of siderophore enterobactin (*entCF*) to chelate and acquire iron were upregulated. Iron is an essential nutrient for the metabolism and growth of bacterial cells, but much attention has been paid during colonization competence and oxidative stress to understand these mechanisms of iron regulation [45]. In this sense, production of growth-inhibitory siderophores with compatible receptor for iron uptake has been studied as a mechanism against plant pathogens [46,47]. Based on the finding that the *asr* gene was upregulated by four-fold, and as diverse studies have confirmed its expression during the response to external acidity and for survival [48], it is probable that the mycelial growth of *S. rolfsii* could cause a lower pH through the production of organic acids that cause oxidative stress in *K. cowanii* Ch1 and generate oxidative stress responses. Upregulated spermidine export genes *mdtIJ* and spermidine N1-acetyltransferase (*speG*) are required probably for stress tolerance in *K. cowanii* Ch1 [49]. Cellular oxidant detoxification and glutathione metabolic processes, as important components of *K. cowanii* Ch1 for stress resistance and motility mechanisms that probably evade stressful conditions, were also upregulated. Important downregulated genes were detected as a response to reduce the number of diverse metabolic pathways for stress homeostasis in *K. cowanii* Ch1 (Table S4).

The gene expression analysis revealed that *K. cowanii* Ch1 had a different response in the presence of VOCs (Figure 6B). Only a set of 35 genes were significantly upregulated and 4 genes were significantly downregulated. Among these differentially expressed genes were *fes*, which encodes the enterobactin esterase enzyme that facilitates intracellular iron release, and genes that encode for siderophore-mediated iron transport (*fepBD*, *tonB*, *feoA*, *entS,* and *efeO*), which indicate the importance of this iron uptake system for enzymatic activities and cell viability in this condition [50]. In fact, the genes that encode for pyruvate formate-lyase (PFL) and pyruvate formate-lyase-activating enzyme (PFL-AE) that utilize iron–sulfur clusters were upregulated by three-fold (Table S5). Both of these enzymes are regulated under anaerobic conditions and play an important role in the supply of acetyl-CoA to the citric acid cycle during anaerobic glycolysis in *E. coli* and other facultative anaerobes [51]. Furthermore, two genes *gatYZ* that encode for D-tagatose 1,6-bisphosphate (TagBP)-specific aldolases and are involved in catabolism of galactitol [52] were upregulated. The upregulated genes related to carbohydrate transport systems provide energy to *K. cowanii* Ch1 and indicate its active metabolism during colonizing competence. Genes related to oxidative stress were upregulated, such as *ibpAB* that encodes a 16 kDa heat shock protein [41] and *trxC* that encodes a thioredoxin, which is a protein with a highly conserved active site sequence [(Cys-Gly-Pro-Cys)], with a key role in maintaining the thiol-disulfide redox potential [53]. An interesting gene that was upregulated was *katG*, which encodes for the catalase–peroxidase KatG, and its catalase activity protects against peroxide-mediated oxidative damage in the cells by catalyzing the conversion of hydrogen peroxide to water and oxygen [54]. In this sense, the interpretation of bubble gas production during microbial interaction (Figure 1D) could be related to the KatG activity; however, the production of hydrogen peroxide by *K. cowanii* Ch1 during colonization competence remains to be investigated to understand its production as in other bacteria, as well as the role of KatG during colonization competence under the influence of VOCs [55]. In fact, in a study where *P. fluorescens* Pf0–1 was exposed to volatiles produced by *Collimonas pratensis*, *Serratia plymuthica*, *Paenibacillus* sp., and *Pedobacter* sp., the catalase was upregulated by >3-fold [56]. Another possibility is that hydrogen peroxide could be produced by *S. rolfsii* in response to volatiles produced by the bacterial strains (Figure 3). This hypothesis can be supported by the research work published in 2000 by Sideri and Georgiou [57], in which they demonstrated that hydrogen peroxide was produced by the phytopathogen *S. rolfsii* during its development and influenced the oxidative stress caused by growth factors such as light and iron. Another source of evidence is a study in which it was demonstrated that reactive oxygen species (ROS) were accumulated in *Sclerotinia sclerotiorum* hyphae cells when they were treated with VOCs produced by *Bacillus* endophyte strains as a mechanism of oxidative stress response [58]. Therefore, the presence of bacterial VOCs during the interaction between *K. cowanii* Ch1 and *S. rolfsii* could be a positive response for the biological processes of bacterial colonizing competence in at least the condition evaluated; however, the transcriptomic analysis was performed during the early stage of interaction, and this could be a limiting factor. Additional studies are necessary to understand this complex interactome and evaluate the potential production of biomolecules with antifungal properties under colonizing competence. In fact, some genes related to antimicrobial defense were upregulated in *K. cowanii* Ch1 (Table S5), like those that encode for antibiotic biosynthesis monooxygenase (QU629_RS21570), polyketide synthase modules, and related proteins, which are crucial in the synthesis of polyketides, an important class of secondary metabolites that provide certain survival advantages to diverse organisms [59]. Furthermore, the gene that encodes for the virulence factor VirK was upregulated by two-fold, which is a component of the chaperone pathways involved in the secretion systems to deliver virulence factors, such as the plasmid-encoded toxin in *E. coli* [60].

The differences in the phenotypical responses of *K. cowanii* Ch1 in the presence of VOCs produced by different bacterial strains (Figure 3), including the fungal responses during the interaction assays (Figure 2), appeared to reflect that the composition and concentrations of these mixtures of VOCs as well as their effects on gene expression (Figure 6) can potently modulate communication signals conserved during colonization competence in microorganisms [37]. Therefore, the evaluation of cell-free filtrates of *K. cowanii* Ch1 obtained at different growth time periods (Figure 5), and the important finding that the cell-free filtrate at 36 h, applied in the microbial interaction, caused mycelial growth inhibition in vitro and in vivo suggest that the VOC composition produced at a specific bacterial growth stage could be a key factor in determining the biological processes observed, indicating the importance of understanding its impact on competitors, particularly due to its ability to compromise cell viability and gene expression to reduce infection in chili fruit tissues. In this sense, marked differences were observed between the VOC profiles at 36 h compared to those at 24 h. Some common chemical compounds with potent antifungal activities that have been studied in other bacteria strains [37] were detected with high relative abundance, including Pyrazine, 2,5-Dimethyl; 1-Hexanol, 2-ethyl-; 3-methyl-1-Butanol; Ethanol; 2,2,4-trimethyl-1,3-pentanediol diisobutyrate; Benzaldehyde; 1-Decanol; 1-Octanol; and 6-Methyl-1-octanol. Finally, and in agreement with other studies, the mixtures of VOCs, produced by diverse bacterial strains isolated under specific conditions, represent an important alternative for the biocontrol of phytopathogens [15,16,17]; therefore, our study proposes a cocktail with antimicrobial properties that contains a combination of the aforementioned chemical compounds detected in the HS-SPME-GC-MS analysis.

## 5. Conclusions

In conclusion, the results of this study demonstrated that, under the presence of bacterial VOCs during microbial interactions, *K. cowanii* Ch1 had better biocontrol ability to reduce mycelial growth in *S. rolfsii* in both in vitro and in vivo assays, likely by disrupting multiple metabolic pathways in *S. rolfsii* leading to compromised cell viability. Furthermore, the transcriptomic analysis revealed important upregulated genes, and also demonstrated an additional mechanism related to the production of polyketides; however, additional experimental research is needed to validate this mechanism. The mixture of VOCs identified at a specific growth stage of *K. cowanii* Ch1 provides future perspectives to enhance its metabolic ability for the potential development of an efficient biocontrol strategy as potent modulators of communication signals to promote the colonization competence in *K. cowanii* Ch1.

## Data Availability

The data presented in this work are available from the corresponding authors upon request.

## Supplementary Materials

The following supporting information can be downloaded at https://www.mdpi.com/article/10.3390/microorganisms13071483/s1: Table S1. Profile of volatile organic compounds detected in bacterial strains using HS-SPME-GC-MS. Table S2. Profile of volatile organic compounds detected in *K. cowanii* Ch1 at 24 h and 36 h of bacterial growth using HS-SPME-GC-MS. Table S3. RNA-Seq analysis from RNA samples. Table S4. Top differentially expressed genes in *K. cowanii* Ch1 during interaction with *S. rolfsii* in absence of VOCs. Table S5. Top differentially expressed genes in *K. cowanii* Ch1 during interaction with *S. rolfsii* in presence of VOCs. Figure S1. Evaluation of synthetic VOCs on interaction of *K. cowanii* Ch1 and *S. rolfsii* using a double-compartment Petri dish chamber. Figure S2. Similarities between samples. Larger values indicate higher similarity between samples. The similarities were calculated using normalized and ‘rlog’ transformed read counts of all genes using DESeq2. Figure S3. Scatter plot to visualize differential gene expression results. Mean transformed read counts of genes in one group are shown on X-axis while those in the other are shown on Y-axis. Red dots represent differentially expressed genes (adjusted *p*-values < 0.05).
