# Supplementary material for: Volatile Metabolome and Transcriptomic Analysis of Kosakonia cowanii Ch1 During Competitive Interaction with Sclerotium rolfsii Reveals New Biocontrol Insights
Source: Microorganisms. 2025 Jun 26;13(7):1483. doi: 10.3390/microorganisms13071483 (PMC12300148; doi:10.3390/microorganisms13071483)
Supplement: Supplementary file 1 [file microorganisms-13-01483-s001.zip › microorganisms-3674291-supplementary.pdf]

Table S1. Profile of Volatile organic compounds detected in the bacterial strains using HS-SPME-GC-MS.

		Relative Peak Area (%)			
**Volatile Organic Compounds (VOCs)**	Retention Time (min)	*K. cowanii*	*P. aroidearum*	*B. tropicus*	*B. altitudinis*
**Ethyl acetate**	8.27	0.29	2.46	0.00	0.00
**Ethanol**	9.87	5.56	1.47	0.00	1.36
**1-Butanol, 3-methyl-**	25.51	4.83	4.02	0.20	4.43
**Pyrazine,methyl-**	28.25	1.15	1.04	0.00	2.24
**Pyrazine, 2,5-Dimethyl**	31.18	6.47	4.99	12.33	13.31
**Cyclooctasiloxane, hexadecamethyl-**	46.30	0.00	1.99	0.00	0.00
**1-Decanol**	52.77	0.00	1.02	0.00	0.00
**Butanoic acid, butyl ester**	62.00	4.54	4.25	0.00	0.00
**Butanoic acid, 1-methylpropyl ester**	63.25	0.00	3.24	0.00	0.00
**2,2,4-trimethyl-1,3-pentanediol diisobutyrate**	63.93	0.00	3.67	0.00	0.00
**1-Decene**	70.82	0.00	4.93	0.00	0.00
**Beta-iso-Methyl ionone**	76.02	0.00	1.18	0.00	0.00
**Isopropyl myristate**	76.38	0.00	1.32	0.00	0.00
**n-Tridecan-1-ol**	77.05	0.00	1.68	0.00	0.00
**Cyclotetradecane**	81.97	0.00	1.11	0.00	0.00
**Nonanoic acid**	82.73	0.00	2.53	6.53	6.72
**Cyclopentaneacetic acid, 3-oxo-2-pentyl-, methyl ester**	85.89	0.00	1.16	0.00	0.00
**2-Ethylhexyl salicylate**	86.90	0.00	5.65	0.00	0.00
**Homosalate**	91.49	0.00	1.93	0.00	0.00
**Phthalic acid, isobutyl octyl ester**	95.25	1.10	1.51	0.00	0.00
**Octacosanal**	95.65	0.00	1.11	0.00	0.00
**Carbon dioxide**	2.13	1.81	0.51	1.10	2.13
**n-Hexane**	4.40	0.00	0.00	1.51	2.29
**Heptane**	4.90	1.07	0.90	2.22	2.78
**2,3-Butanedione**	11.55	2.58	0.30	10.38	2.24
**1-butanol**	21.13	1.42	0.55	1.30	1.34
**1,3-Diazine**	24.15	0.48	0.00	1.56	2.40
**1-Pentanol**	24.61	0.00	0.00	3.78	0.00
**Acetoin**	28.44	13.52	0.25	32.77	11.90
**Pyrazine, 2-ethyl-5-methyl-**	33.84	0.43	0.32	1.19	2.68
**Phenylethyl Alcohol**	61.53	1.99	0.22	1.06	0.55
**Octanoic acid**	74.86	0.68	0.40	2.35	2.46
**Piperonal**	81.38	0.00	0.00	1.17	2.14
**n-Decanoic acid**	84.87	0.00	0.00	1.67	2.67
**Acetaldehyde**	4.78	0.54	0.00	0.00	1.07
**Acetone**	5.99	0.61	0.48	0.68	1.04
**Butanal, 3-methyl-**	8.69	0.23	0.81	0.00	1.67
**Disulfide, dimethyl**	16.14	1.35	0.72	0.22	1.03
**1-Hexanol**	32.23	0.20	0.11	0.51	1.17

**Pyrazine, trimethyl-**	34.37	0.55	0.53	0.98	1.22
**Acetic acid**	36.04	0.21	0.17	0.18	2.02
**4-Heptanol, 2,6-dimethyl-**	37.69	0.00	0.00	0.00	1.10
**Benzaldehyde**	39.47	0.94	0.39	0.00	1.99
**1-Octanol**	41.61	1.62	0.73	0.00	1.62
**Butanoic acid, 3-methyl-**	45.78	0.00	0.00	0.00	3.54
**2-Nonanone**	35.00	1.32	0.00	0.00	0.00
**1-Hexanol, 2-ethyl-**	39.46	2.22	0.00	0.51	1.15
**2-Undecanone**	44.34	1.12	0.00	0.00	0.00
**Cyclodecane**	53.11	3.10	0.00	0.00	0.00
**2-Tridecanone**	57.17	1.25	0.00	0.00	0.00
**Propanoic acid, 2-methyl-, 2,2-dimethyl-1-(2-hydroxy-1-methylethyl) propyl ester**	63.49	2.12	0.00	0.00	0.00
**2,4,7,9-Tetramethyl-5-decyn-4,7-diol**	78.39	1.90	0.00	0.00	0.00
**Dodecanoic acid, 3-hydroxy-**	85.69	1.13	0.00	0.00	0.00

Table S2. Profile of Volatile organic compounds detected in *K. cowanii* Ch1 at 24 h and 36 h of bacterial growth using HS-SPME-GC-MS.

		**Relative Peak Area (%)**	
**Volatic Organic Compound (VOC's)**	Retention time (min)	24 h	36 h
**Ethanol**	14.76	5.56	3.91
**1-Butanol, 3-methyl-**	31.08	4.83	4.5
**Pyrazine,methyl-**	33.87	1.15	1.03
**Pyrazine, 2,5-Dimethyl**	36.87	6.47	9.82
**1-Decanol**	63.79	0	2.88
**Butanoic acid, butyl ester**	61.66	4.54	0
**2,2,4-trimethyl-1,3-pentanediol diisobutyrate**	75.95	0	3.42
**Isopropyl myristate**	84.81	0	0.81
**Phthalic acid, isobutyl octyl ester**	95.254	1.1	0
**Carbon dioxide**	7.2	1.81	2.81
**Heptane**	11.55	1.07	0
**2,3-Butanedione**	17.82	2.15	0.15
**1-butanol**	27.62	1.74	0.91
**Acetoin**	35.04	13.52	1.88
**Phenylethyl Alcohol**	73.57	1.99	0.35
**Acetaldehyde**	8.46	0.54	1.08
**Butanal, 3-methyl-**	15.22	0.23	1.96
**Disulfide, dimethyl**	23.31	1.35	0.58
**Benzaldehyde**	46.3	0.94	3.03
**1-Octanol**	48.17	1.62	2.87
**2-Nonanone**	41.3	1.32	0.88

**1-Hexanol, 2-ethyl-**	44.96	2.22	4.81
**2-Undecanone**	44.34	1.12	0
**Cyclodecane**	53.11	3.1	0
**2-Tridecanone**	57.17	1.25	0
**Propanoic acid, 2-methyl-, 2,2-dimethyl-1-(2-hydroxy-1-methylethyl)propyl ester**	72.98	2.12	0.86
**2,4,7,9-Tetramethyl-5-decyn-4,7-diol**	84.42	1.9	0.26
**Dodecanoic acid, 3-hydroxy-**	85.69	1.13	0
**Nonanal**	41.64	0	1.74
**1-Hexene, 3,3-dimethyl-**	48.42	0	1.25
**6-Methyl-1-octanol**	48.72	0	2.41
**Tetrahydrogeranyl formate**	51.22	0	1.45
**1H-Tetrazole, 1,5-dimethyl-**	51.57	0	1.64
**2-Furanmethanol**	52.68	0	1.11
**3-Cyclohexene-1-methanol**	57.34	0	1.56
**Benzyl alcohol**	70.82	0.54	1.24

Table S3. RNA-Seq analysis from RNA samples.

Sample Name	M Seqs	% GC	% Reads PF	Reads Removed (%)	% Aligned	% Dups	% rRNA	% Assigned	M Assigned	Numbers of genes detected
Treatment_kosakonia_1_TK1	52.7	45%	99.0%	0.2	24.3%	63.9%	3.59%	88.5%	11.2	4434
Treatment_kosakonia_2_TK2	46.1	50%	98.8%	0.3	44.2%	65.3%	3.37%	88.7%	17.8	4438
Treatment_kosakonia_VOCS_1_TKV1	48.7	53%	98.7%	0.3	93.0%	61.0%	2.69%	90.0%	40.2	4446
Treatment_kosakonia_VOCS_2_TKV2	42.3	53%	98.7%	0.4	86.7%	61.8%	3.08%	88.7%	32.0	4440
control_kosakonia_1_CK1	48.4	53%	98.4%	0.3	96.0%	68.0%	3.04%	90.5%	41.2	4444
control_kosakonia_2_CK2	47.0	53%	98.6%	0.3	98.9%	66.9%	2.65%	90.0%	41.2	4443

Table S4. Top differentially expressed genes in *K. cowanii* Ch1 during interaction with *S. rolfsii* in absence of VOCs.

Gene	ID	Description	Log2FoldChange
QU629_RS04135	QU629_RS04135	Endonuclease IV	5.813828467
QU629_RS21790	QU629_RS21790	Zinc ABC transporter, permease protein ZnuB	5.686304244
QU629_RS21795	QU629_RS21795	Zinc ABC transporter, substrate-binding protein ZnuA	5.668940418
QU629_RS01540	QU629_RS01540	Multiple stress resistance protein BhsA	5.406536877
QU629_RS16930	QU629_RS16930	2-Oxobutyrate oxidase	5.314873984
ibpB	QU629_RS08580	16 kDa heat shock protein B	5.161516989
yhcN	QU629_RS12965	Probable exported protein YPO3518	5.124143803
ibpA	QU629_RS08585	16 kDa heat shock protein A	4.491736004
gap	QU629_RS20245	NAD-dependent glyceraldehyde-3-phosphate dehydrogenase	4.373717339
QU629_RS04595	QU629_RS04595	Transcriptional regulator YqjI	4.289350676
mdtJ	QU629_RS00190	Spermidine export protein MdtJ	4.226187686
QU629_RS14015	QU629_RS14015	Enterobactin esterase	4.12180492
asr	QU629_RS00170	Acid shock protein precursor	4.076390679
QU629_RS20110	QU629_RS20110	3-oxoacyl-[acyl-carrier-protein] synthase	4.046607462
fhuF	QU629_RS20590	Ferric reductase	3.997824411
mdtI	QU629_RS00185	Spermidine export protein MdtI	3.991841233
degP	QU629_RS06980	HtrA protease/chaperone protein	3.91158984
QU629_RS02135	QU629_RS02135	dehydratase	3.907931239
exbB	QU629_RS04865	TonB-ExbBD energy transducing system	3.845612374
QU629_RS22020	QU629_RS22020	Phenazine biosynthesis protein PhzF like	3.801701856
entC	QU629_RS13980	Isochorismate synthase	3.782713484
spy	QU629_RS00840	Spheroplast protein Y	3.763658189
mgtA	QU629_RS12445	Mg(2+) transport ATPase, P-type	3.64345109
QU629_RS10065	QU629_RS10065	Phenolic acid decarboxylase	3.641014858
QU629_RS13845	QU629_RS13845	Siderophore achromobactin ABC transporter, permease protein	3.468290061
kdpA	QU629_RS19735	Potassium-transporting ATPase A chain	3.411039208
rpmE	QU629_RS17815	LSU ribosomal protein L31p	3.287944719
QU629_RS20300	QU629_RS20300	2-oxoglutarate/malate translocator	3.270279319
exbD	QU629_RS04870	TonB-ExbBD energy transducing system	3.165936765
ahpC	QU629_RS13750	Alkyl hydroperoxide reductase protein C	3.157272575
kdpB	QU629_RS19740	Potassium-transporting ATPase B chain	3.126541533
fepD	QU629_RS13995	Ferric enterobactin transport system permease protein FepD	3.093925432
QU629_RS01865	QU629_RS01865	Transcriptional regulator LysR family	3.063039628
znuC	QU629_RS16225	Zinc ABC transporter, ATP-binding protein ZnuC	3.001216976

gntX	QU629_RS09800	Competence protein F homolog	2.990090654
fepB	QU629_RS13985	Ferric enterobactin-binding periplasmic protein FepB	2.989072191
azuC	QU629_RS19115	Stress response protein AzuC	2.979672329
QU629_RS01240	QU629_RS01240	Transcriptional regulator, AcrR family	2.963219562
nrdH	QU629_RS11320	Glutaredoxin-like protein NrdH	2.924228323
QU629_RS16935	QU629_RS16935	Methionine ABC transporter substrate-binding protein	2.908772815
grpE	QU629_RS11790	Heat shock protein GrpE	2.904571587
phoP	QU629_RS01420	Transcriptional regulatory protein PhoP	2.869201653
QU629_RS16945	QU629_RS16945	Methionine ABC transporter permease protein	2.828729751
QU629_RS01110	QU629_RS01110	Transcriptional regulator HxlR family	2.800283272
QU629_RS16940	QU629_RS16940	Methionine ABC transporter ATP-binding protein	2.768243224
edd	QU629_RS16260	Phosphogluconate dehydratase	2.766234859
clpB	QU629_RS12040	Chaperone protein ClpB (ATP-dependent unfoldase)	2.762010024
grcA	QU629_RS10465	Autonomous glycyl radical cofactor	2.71929221
QU629_RS13925	QU629_RS13925	Hydroxycarboxylate dehydrogenase	2.673667612
QU629_RS12815	QU629_RS12815	Sensory box/GGDEF family protein	2.659469824
feoA	QU629_RS09770	Ferrous iron transporter-associated protein FeoA	2.651672404
QU629_RS00180	QU629_RS00180	Diguanylate cyclase/phosphodiesterase	2.62981541
punC	QU629_RS00520	Inner membrane transport protein YdhC	2.619276479
QU629_RS12280	QU629_RS12280	UPF0053 inner membrane protein YtfL	2.615674407
ahpF	QU629_RS13745	Alkyl hydroperoxide reductase protein F	2.605578297
QU629_RS20385	QU629_RS20385	Ferrichrome-iron receptor	2.58798263
tonB	QU629_RS06685	TonB-ExbBD energy transducing system	2.580075203
QU629_RS04345	QU629_RS04345	Osmoprotectant ABC transporter inner membrane protein YehW	2.567932182
feoB	QU629_RS09765	Ferrous iron transporter FeoB	2.555024664
flgC	QU629_RS11475	Flagellar basal-body rod protein FlgC	2.552953701
marA	QU629_RS22000	Multiple antibiotic resistance protein MarA	2.495516326
nrdI	QU629_RS11315	Ribonucleotide reduction protein NrdI	2.487998855
QU629_RS20365	QU629_RS20365	Iron compound ABC transporter, permease protein	2.479046381
phoQ	QU629_RS01425	Sensor histidine kinase PhoQ	2.476953707
fabF	QU629_RS20115	3-oxoacyl-[acyl-carrier-protein] synthase, KASII	2.461938868
QU629_RS16765	QU629_RS16765	Protein QmcA	2.460277649
znuB	QU629_RS16220	Zinc ABC transporter, permease protein ZnuB	2.453748263
QU629_RS04240	QU629_RS04240	Inner membrane protein YohK	2.449583597

dnaK	QU629_RS07580	Chaperone protein DnaK	2.437022654
QU629_RS20360	QU629_RS20360	Iron compound ABC transporter, permease protein	2.426937579
mlaD	QU629_RS13120	Phospholipid ABC transporter substrate-binding protein MlaD	2.388739368
hslV	QU629_RS17835	ATP-dependent protease subunit HslV	2.377981957
QU629_RS09545	QU629_RS09545	Heat shock protein 10 kDa family chaperone	2.371853457
ybeD	QU629_RS13655	Proposed lipoate regulatory protein YbeD	2.371058258
dnaJ	QU629_RS07575	Chaperone protein DnaJ	2.360203869
marB	QU629_RS21995	Multiple antibiotic resistance protein MarB	2.34557562
QU629_RS19045	QU629_RS19045	BarA-associated response regulator UvrY	2.321308638
QU629_RS06235	QU629_RS06235	LysR family transcriptional regulator YdcI	2.316964659
dacC	QU629_RS02795	D-alanyl-D-alanine carboxypeptidase	2.314414281
tatE	QU629_RS13675	Twin-arginine translocation protein TatE	2.313124146
QU629_RS13915	QU629_RS13915	5-methylthioribose ABC transporter, permease protein	2.305813691
QU629_RS02160	QU629_RS02160	CFA/I fimbrial major subunit	2.301688903
feoC	QU629_RS09760	Ferrous iron-sensing transcriptional regulator FeoC	2.29966565
yceG	QU629_RS01615	Murein endolytic transglycosylase MltG	2.286341522
groL	QU629_RS09550	Heat shock protein 60 kDa family chaperone	2.269502812
fliF	QU629_RS11625	Flagellar M-ring protein FliF	2.23542324
QU629_RS04535	QU629_RS04535	Multidrug efflux system MdtABC-TolC	2.227656893
fhuA	QU629_RS07035	Ferric hydroxamate outer membrane receptor	2.225299345
soxR	QU629_RS09255	Redox-sensitive transcriptional activator SoxR	2.220866651
QU629_RS04590	QU629_RS04590	Methyl-accepting chemotaxis sensor protein STM3216	2.188933377
QU629_RS13885	QU629_RS13885	ABC transporter, substrate-binding protein	2.174863957
QU629_RS01545	QU629_RS01545	HTH-type transcriptional repressor ComR	2.151297978
QU629_RS13920	QU629_RS13920	5-methylthioribose ABC transporter	2.110830149
speG	QU629_RS00075	Spermidine N1-acetyltransferase	2.097820856
QU629_RS01215	QU629_RS01215	Outer membrane protein V	2.052102872
zntR	QU629_RS21395	Transcriptional regulator ZntR	2.025948794
mlaE	QU629_RS13115	Phospholipid ABC transporter permease protein MlaE	2.019748472
norR	QU629_RS21645	Anaerobic nitric oxide reductase transcription regulator NorR	1.956850227
QU629_RS13880	QU629_RS13880	ABC transporter, permease protein	1.944554792
msrA	QU629_RS12290	Peptide-methionine (S)-S-oxide reductase	1.921574941
QU629_RS07965	QU629_RS07965	Inner membrane protein YiaH	1.919182969
QU629_RS04235	QU629_RS04235	Cytidine deaminase	1.909849644

entF	QU629_RS14010	Enterobactin synthetase component F	1.907402469
QU629_RS02940	QU629_RS02940	Sugar phosphatase YbiV	1.893464894
QU629_RS07505	QU629_RS07505	Putative outer membrane lipoprotein	1.846972786
dps	QU629_RS03040	Threonine/homoserine exporter RhtA	1.828070961
QU629_RS12260	QU629_RS12260	2',3'-cyclic-nucleotide 2'-phosphodiesterase	1.816743881
lon	QU629_RS17145	ATP-dependent protease La	1.813940307
nfuA	QU629_RS09805	[4Fe-4S] cluster carrier protein NfuA	1.813561426
QU629_RS02780	QU629_RS02780	Multidrug efflux pump MdfA/Cmr (of MFS type)	1.808931862
QU629_RS01120	QU629_RS01120	Transcriptional regulator, AraC family	1.789350924
cydX	QU629_RS19630	Cytochrome d ubiquinol oxidase subunit X	1.789001509
QU629_RS20550	QU629_RS20550	Organic hydroperoxide resistance transcriptional regulator	1.788678098
cysC	QU629_RS15185	Adenylylsulfate kinase	1.771140639
cysD	QU629_RS15175	Sulfate adenylyltransferase subunit 2	1.762786377
QU629_RS04970	QU629_RS04970	Acetolactate synthase, catabolic	1.746203682
metA	QU629_RS08890	Homoserine O-succinyltransferase	1.740870021
creB	QU629_RS07665	Response regulator CreB of two-component signal transduction system CreBC	1.740726098
QU629_RS20380	QU629_RS20380	Transcriptional regulator, AraC family	1.736220551
asd	QU629_RS09645	Phosphatidylserine decarboxylase	1.735830019
pabC	QU629_RS01620	Aminodeoxychorismate lyase	1.725378755
QU629_RS00565	QU629_RS00565	Major outer membrane lipoprotein Lpp	1.71784913
QU629_RS09180	QU629_RS09180	Periplasmic chorismate mutase I precursor	1.714975046
flgE	QU629_RS11485	Flagellar hook protein FlgE	1.709971957
QU629_RS14735	QU629_RS14735	DNA-binding transcriptional dual regulator	1.7074971
msrP	QU629_RS12880	Protein-methionine-sulfoxide reductase catalytic subunit MsrP	1.701584
nrdG	QU629_RS12430	Ribonucleotide reductase of class III (anaerobic)	1.694793611
QU629_RS02335	QU629_RS02335	L-rhamnose operon transcriptional activator RhaR	1.693999219
traT	QU629_RS15625	IncF plasmid conjugative transfer surface	1.684327426
QU629_RS00970	QU629_RS00970	Organic hydroperoxide resistance transcriptional regulator	1.674632279
QU629_RS10875	QU629_RS10875	Uncharacterized MFS-type transporter	1.668439329
QU629_RS20095	QU629_RS20095	Transcriptional regulator YafC	1.658747414
bamC	QU629_RS11030	Outer membrane beta-barrel assembly	1.657655707
marR	QU629_RS22005	Multiple antibiotic	1.656692314
gstA	QU629_RS00400	Glutathione S-transferase	1.655533229

gapA	QU629_RS00935	NAD-dependent glyceraldehyde-3-phosphate dehydrogenase	1.655493719
QU629_RS12705	QU629_RS12705	Two-component system sensor histidine kinase	1.655165227
QU629_RS04295	QU629_RS04295	Uncharacterized oxidoreductase YohF	1.642707348
thiB	QU629_RS07400	Thiamin ABC transporter, substrate-binding component	1.639364975
fepC	QU629_RS14005	Ferric enterobactin transport ATP-binding protein FepC	1.63889561
trxC	QU629_RS10450	Thioredoxin 2	1.61089708
QU629_RS10915	QU629_RS10915	Two-component transcriptional response regulator	1.601550273
soxS	QU629_RS09250	DNA-binding transcriptional dual regulator SoxS	1.597439856
QU629_RS13940	QU629_RS13940	Oxidoreductase, short-chain dehydrogenase	1.590944596
fliI	QU629_RS11640	Flagellum-specific ATP synthase FliI	1.585194449
QU629_RS15145	QU629_RS15145	Glycine cleavage system transcriptional activator GcvA	1.581224701
exuR	QU629_RS13490	Hexuronate utilization operon transcriptional repressor	1.570989691
nrdD	QU629_RS12435	Ribonucleotide reductase of class III (anaerobic)	1.570021361
QU629_RS17210	QU629_RS17210	2-dehydropantoate 2-reductase	1.552598599
QU629_RS21270	QU629_RS21270	Thiamin-phosphate pyrophosphorylase	1.551439082
sucA	QU629_RS19660	2-oxoglutarate dehydrogenase E1 component	1.535027203
kdpC	QU629_RS19745	Potassium-transporting ATPase C chain	1.534829486
QU629_RS11440	QU629_RS11440	Alkyl hydroperoxide reductase subunit C	1.532235729
ytfE	QU629_RS12235	Repair of Iron Centers di-iron protein	1.531979055
cysG	QU629_RS21185	Precorrin-2 oxidase	1.529050437
QU629_RS08385	QU629_RS08385	Aldo/keto reductase	1.528716452
QU629_RS06155	QU629_RS06155	Benzoate transport protein	1.522720281
ydfG	QU629_RS00045	NADP-dependent 3-hydroxy acid dehydrogenase YdfG	1.520663086
greA	QU629_RS13180	Transcription elongation factor GreA	1.519313717
adhP	QU629_RS05655	Alcohol dehydrogenase	1.502003915
iscR	QU629_RS10635	Iron-sulfur cluster regulator IscR	1.49946484
htpG	QU629_RS16840	Chaperone protein HtpG	1.497113218
mutM	QU629_RS08215	Formamidopyrimidine-DNA glycosylase	1.496768603
bamE	QU629_RS11775	Outer membrane beta-barrel assembly protein BamE	1.495074462
mtnA	QU629_RS13900	S-methyl-5-thioribose-1-phosphate isomerase	1.494959202
slyA	QU629_RS00430	Transcriptional regulator SlyA	1.49260356
efeU	QU629_RS01960	Ferrous iron transport permease EfeU	1.489949209
QU629_RS03245	QU629_RS03245	Oxidoreductase	1.482462237
zntB	QU629_RS06380	Zinc transport protein ZntB	1.480373168

yieE	QU629_RS04140	Transcriptional regulator YeiE	1.475194744
QU629_RS20655	QU629_RS20655	4-carboxymuconolactone decarboxylase domain	1.474496303
QU629_RS00350	QU629_RS00350	Inner membrane protein YdgK	1.459419173
fhuB	QU629_RS07020	Ferric hydroxamate ABC transporter	1.45772672
QU629_RS20650	QU629_RS20650	Transcriptional regulator, GntR family domain	1.455828195
QU629_RS12255	QU629_RS12255	Redox-sensing transcriptional regulator QorR	1.445615559
robA	QU629_RS07675	DNA-binding transcriptional dual regulator	1.441931939
mqo	QU629_RS04000	Malate:quinone oxidoreductase	1.435871899
bamD	QU629_RS12025	Outer membrane beta-barrel assembly	1.421175866
QU629_RS16085	QU629_RS16085	Uncharacterized inner membrane transporter YedA	1.42101358
QU629_RS15240	QU629_RS15240	Transcriptional regulator STM2912	1.418904667
efeB	QU629_RS01950	Ferrous iron transport peroxidase EfeB	1.414031183
dapA	QU629_RS11025	4-hydroxy-tetrahydrodipicolinate synthase	1.405706111
QU629_RS15790	QU629_RS15790	NAD(P)H-flavin oxidoreductase	1.398454202
rsxC	QU629_RS00365	Electron transport complex protein	1.395352513
QU629_RS17440	QU629_RS17440	TonB-dependent hemin	1.388579314
tpx	QU629_RS06430	Thiol peroxidase	1.379568615
QU629_RS10870	QU629_RS10870	Hypothetical ABC transport system, periplasmic component	1.379316789
QU629_RS15490	QU629_RS15490	IncF plasmid conjugative transfer pilus	1.375076236
nrdE	QU629_RS11310	Ribonucleotide reductase of class Ib (aerobic)	1.374452611
QU629_RS00800	QU629_RS00800	Membrane-bound metal-dependent hydrolase YdjM	1.374137402
fhuC	QU629_RS07030	Ferric hydroxamate ABC transporter	1.356843446
tolA	QU629_RS19605	Methyl-accepting chemotaxis sensor	1.348709229
purB	QU629_RS01415	Adenylosuccinate lyase	1.339446354
QU629_RS04530	QU629_RS04530	Multidrug efflux system MdtABC-TolC	1.337128687
QU629_RS13455	QU629_RS13455	Inner membrane protein YqjK	1.332792943
QU629_RS10280	QU629_RS10280	Trehalase	1.331782116
QU629_RS07850	QU629_RS07850	YoeB toxin protein	1.314454386
QU629_RS03530	QU629_RS03530	Long-chain fatty acid transport protein	1.278786374
QU629_RS20660	QU629_RS20660	Methyl-accepting chemotaxis sensor/transducer protein	1.270518229
gshB	QU629_RS05205	Glutathione synthetase	1.263964223
QU629_RS06505	QU629_RS06505	Enoyl-[acyl-carrier-protein] reductase	1.262367987
ruvC	QU629_RS16195	Crossover junction endodeoxyribonuclease RuvC	1.26067089
copA	QU629_RS16800	Lead, cadmium, zinc and mercury transporting ATPase	1.25361867

QU629_RS15745	QU629_RS15745	Transcriptional regulator, HxlR family	1.247181002
psiF	QU629_RS17325	Phosphate starvation-inducible protein PsiF	1.240993855
dinF	QU629_RS09095	DNA-damage-inducible protein F	1.216262938
QU629_RS10300	QU629_RS10300	Transcriptional regulator YhjC, LysR family	1.215426947
cysG	QU629_RS15170	Precorrin-2 oxidase	1.189158945
QU629_RS07595	QU629_RS07595	L-Proline/Glycine betaine transporter ProP	1.183436301
rsxD	QU629_RS00370	Electron transport complex protein	1.15338298
QU629_RS05370	QU629_RS05370	Transcriptional regulator, RpiR family	1.123071369
QU629_RS03005	QU629_RS03005	Inner-membrane proton/drug antiporter (MSF type) of tripartite multidrug efflux system	-1.431833061
QU629_RS05070	QU629_RS05070	Tricarboxylate transport transcriptional regulator TctD	-1.432239508
ldtB	QU629_RS02970	Biosintesis de la pared celular	-1.432495145
QU629_RS12400	QU629_RS12400	D-glucosaminate-6-phosphate ammonia-lyase related protein	-1.44197416
wzzE	QU629_RS18290	Lipopolysaccharide biosynthesis protein	-1.445694612
phoC	QU629_RS00900	Acid phosphatase	-1.447925114
QU629_RS03505	QU629_RS03505	Putative inner membrane protein	-1.451795838
QU629_RS05525	QU629_RS05525	Sensory box histidine kinase/response regulator	-1.483223108
QU629_RS17030	QU629_RS17030	Dihydrolipoamide dehydrogenase of acetoin dehydrogenase	-1.486765254
glpK	QU629_RS17865	Glycerol kinase	-1.493567295
QU629_RS05685	QU629_RS05685	Aerotaxis sensor receptor protein	-1.495259903
QU629_RS09515	QU629_RS09515	Cytochrome c-type biogenesis protein DsbD	-1.496095358
dppA	QU629_RS07825	Dipeptide ABC transporter	-1.503692536
tdh	QU629_RS08130	L-threonine 3-dehydrogenase	-1.5169583
QU629_RS09205	QU629_RS09205	Inositol-1-monophosphatase	-1.519010931
mtfA	QU629_RS14640	Protein MtfA	-1.534312182
wzb	QU629_RS14215	Low molecular weight protein-tyrosine-phosphatase	-1.550901318
QU629_RS04920	QU629_RS04920	Glutathione S-transferase	-1.56542489
phoH	QU629_RS01945	Phosphate starvation-inducible protein PhoH,	-1.570205304
flgC	QU629_RS01745	Flagellar basal-body rod protein FlgC	-1.602017262
QU629_RS04745	QU629_RS04745	ADP-ribose pyrophosphatase	-1.607036085
QU629_RS04290	QU629_RS04290	Small membrane protein YohP	-1.610805117
argG	QU629_RS13220	Argininosuccinate synthase	-1.612336633
phoR	QU629_RS17260	Phosphate regulon sensor protein PhoR	-1.616643897
ilvC	QU629_RS18215	Ketol-acid reductoisomerase	-1.623177171

cydB	QU629_RS06105	Cytochrome d ubiquinol oxidase subunit II	-1.624335615
QU629_RS04405	QU629_RS04405	Maltose/maltodextrin transport ATP-binding protein MalK	-1.626185543
ydcK	QU629_RS06185	Putative transferase clustered with tellurite resistance proteins TehA	-1.628928903
QU629_RS03515	QU629_RS03515	Bactoprenol-linked glucose translocase	-1.630675125
QU629_RS18700	QU629_RS18700	Putative membrane protein YchH	-1.635125855
QU629_RS20525	QU629_RS20525	Methyl-accepting chemotaxis protein I	-1.645732355
QU629_RS05060	QU629_RS05060	Putative inner membrane protein YqgA	-1.647335008
argH	QU629_RS17755	Argininosuccinate lyase	-1.658708725
gntT	QU629_RS09810	High-affinity gluconate transporter GntT	-1.667642771
tamA	QU629_RS12295	Outer membrane component of TAM transport system	-1.675223743
pphA	QU629_RS16320	Serine/threonine protein phosphatase	-1.682083473
QU629_RS07900	QU629_RS07900	2-ketogluconate transporter	-1.68378307
fumA	QU629_RS00280	Fumarate hydratase class I	-1.708699836
flgB	QU629_RS01750	Flagellar basal-body rod protein FlgB	-1.71125348
potC	QU629_RS01455	Spermidine/putrescine import ABC transporter permease protein	-1.715178698
QU629_RS18475	QU629_RS18475	Transcriptional activator MetR	-1.716865178
cspE	QU629_RS13690	Cold shock protein of CSP family	-1.721791012
QU629_RS13575	QU629_RS13575	Glutamate/aspartate ABC transporter	-1.725961069
paoA	QU629_RS17470	Periplasmic aromatic aldehyde oxidoreductase	-1.728590706
traF	QU629_RS09110	Putative outer membrane or exported protein	-1.729011309
argB	QU629_RS17760	N-acetylglutamate kinase	-1.731006577
QU629_RS14575	QU629_RS14575	FMN-dependent NADH-azoreductase	-1.731662485
malQ	QU629_RS09815	4-alpha-glucanotransferase (amylomaltase)	-1.733358249
potD	QU629_RS01460	Spermidine/putrescine import ABC transporter substrate-binding protein	-1.736430453
QU629_RS14660	QU629_RS14660	Transcriptional repressor of the lac operon	-1.741096108
fadI	QU629_RS03535	3-ketoacyl-CoA thiolase	-1.748063515
hutX	QU629_RS17455	Putative heme iron utilization protein	-1.751100664
gmd	QU629_RS14255	GDP-mannose 4,6-dehydratase	-1.754533888
QU629_RS20625	QU629_RS20625	Uncharacterized GGDEF domain protein YneF	-1.769383908
QU629_RS21285	QU629_RS21285	NADH pyrophosphatase	-1.773411102
wcaE	QU629_RS14245	Colanic acid biosynthesis glycosyl transferase	-1.777870974
QU629_RS17355	QU629_RS17355	Inner membrane protein YaiY	-1.792920846

patD	QU629_RS06090	4-aminobutyraldehyde dehydrogenase	-1.798242443
QU629_RS09325	QU629_RS09325	Response regulator protein	-1.807005895
ycgZ	QU629_RS18920	Probable two-component-system connector protein YcgZ	-1.824938366
QU629_RS09275	QU629_RS09275	Myo-inositol 2-dehydrogenase	-1.830437116
glnK	QU629_RS17085	Nitrogen regulatory protein P-II, GlnK	-1.839943751
QU629_RS04880	QU629_RS04880	Uncharacterized oxidoreductase YghA	-1.849772602
QU629_RS19210	QU629_RS19210	Methyl-accepting chemotaxis protein II	-1.852752332
QU629_RS10430	QU629_RS10430	Putative outer membrane lipoprotein	-1.8538553
ompW	QU629_RS06660	Outer membrane protein W precursor	-1.857381108
malP	QU629_RS09820	Maltodextrin phosphorylase	-1.863154589
QU629_RS09510	QU629_RS09510	Transcriptional regulator YjdC	-1.865446142
aaeR	QU629_RS12925	Transcriptional regulator AaeR, LysR family	-1.871310514
rhaS	QU629_RS17980	Predicted L-rhamnose ABC transporter	-1.888997034
yjfF	QU629_RS12330	Galactofuranose ABC transporter, permease protein 2	-1.898512846
QU629_RS01345	QU629_RS01345	Methyl-accepting chemotaxis sensor/transducer protein	-1.900697796
QU629_RS05565	QU629_RS05565	Chemotaxis regulator - transmits chemoreceptor signals to flagellar motor components CheY	-1.948434679
QU629_RS14210	QU629_RS14210	Low molecular weight protein-tyrosine-phosphatase	-1.949472838
QU629_RS08410	QU629_RS08410	Uncharacterized zinc-type alcohol dehydrogenase-like protein YbdR	-1.957149195
QU629_RS04580	QU629_RS04580	Putrescine aminotransferase	-1.961025883
gltJ	QU629_RS13580	Glutamate/aspartate ABC transporter	-1.966386124
QU629_RS11750	QU629_RS11750	Type I secretion membrane fusion protein, HlyD family	-1.970183138
artJ	QU629_RS02690	Arginine ABC transporter, substrate-binding protein ArtJ	-1.970916778
QU629_RS18940	QU629_RS18940	Flagellin FliC	-1.978717959
QU629_RS05705	QU629_RS05705	Thiamine pyrophosphate-requiring protein PA2108	-1.981371229
argC	QU629_RS17765	N-acetyl-gamma-glutamyl-phosphate reductase	-1.98977371
garD	QU629_RS13395	D-galactarate dehydratase	-1.993098328
putP	QU629_RS01975	Transcriptional repressor of PutA and PutP	-1.994228792
QU629_RS03000	QU629_RS03000	Efflux transport system, outer membrane factor (OMF) lipoprotein	-1.995455362
ycaC	QU629_RS09115	Nicotinamidase family protein YcaC	-2.021010283
QU629_RS01920	QU629_RS01920	Anaerobic respiratory reductase chaperone	-2.032039357
QU629_RS17940	QU629_RS17940	Superoxide dismutase	-2.038004916
QU629_RS21775	QU629_RS21775	PTS system, glucitol/sorbitol-specific IIC component	-2.056185285

QU629_RS17860	QU629_RS17860	Glycerol kinase	-2.065007828
QU629_RS00660	QU629_RS00660	Arginine ABC transporter, substrate-binding protein ArtJ	-2.068273119
QU629_RS21245	QU629_RS21245	Cytochrome c-type biogenesis protein DsbD	-2.073232205
QU629_RS08045	QU629_RS08045	PTS system, mannitol-specific IIC component	-2.07987993
bcsQ	QU629_RS10365	Cellulose biosynthesis protein BcsQ	-2.098319477
pstC	QU629_RS08715	Phosphate ABC transporter, permease protein PstC	-2.101956387
wcaF	QU629_RS14250	Colanic acid biosynthesis acetyltransferase	-2.110449281
traB	QU629_RS15510	IncF plasmid conjugative transfer pilus assembly protein	-2.128092752
flhD	QU629_RS11700	Flagellar transcriptional activator FlhD	-2.135564878
QU629_RS14935	QU629_RS14935	4-deoxy-L-threo-5-hexosulose-uronate ketol-isomerase	-2.170501611
potA	QU629_RS01445	Spermidine/putrescine import ABC transporter ATP-binding protein PotA	-2.17773531
srlB	QU629_RS21770	PTS system, glucitol/sorbitol-specific IIB component	-2.185982776
rhaD	QU629_RS17975	Rhamnulose-1-phosphate aldolase	-2.198290451
QU629_RS09235	QU629_RS09235	ABC transporter, permease protein 2	-2.223986629
QU629_RS06100	QU629_RS06100	DNA-binding transcriptional regulator, MocR family	-2.231251675
QU629_RS02065	QU629_RS02065	Diguanylate cyclase	-2.239906863
QU629_RS19335	QU629_RS19335	Endonuclease/exonuclease/phosphatase family protein	-2.254945045
ugpE	QU629_RS09905	Glycerol-3-phosphate ABC transporter	-2.261986789
ppsA	QU629_RS00655	Phosphoenolpyruvate synthase	-2.265251151
fadA	QU629_RS22040	3-ketoacyl-CoA thiolase	-2.267040765
QU629_RS21135	QU629_RS21135	Para-aminobenzoate synthase	-2.267736422
QU629_RS04720	QU629_RS04720	TRAP-type transport system,	-2.272814086
argF	QU629_RS12475	Ornithine carbamoyltransferase	-2.278895773
rhaR	QU629_RS17955	L-rhamnose operon transcriptional activator RhaR	-2.313088953
QU629_RS18495	QU629_RS18495	ABC transporter, ATP-binding protein	-2.315924004
QU629_RS21250	QU629_RS21250	2-iminoacetate synthase	-2.320671276
malZ	QU629_RS21000	Maltodextrin glucosidase	-2.339760341
gltK	QU629_RS13585	Glutamate/aspartate ABC transporter	-2.341815593
ompF	QU629_RS02370	Outer membrane porin OmpF	-2.348623702
QU629_RS20750	QU629_RS20750	ABC transporter, permease protein	-2.356044579
ugpA	QU629_RS09910	Glycerol-3-phosphate ABC transporter	-2.359540275
QU629_RS02950	QU629_RS02950	Transcriptional regulator, LacI family	-2.360108668
QU629_RS09230	QU629_RS09230	ABC transporter, permease protein	-2.364774563

QU629_RS02985	QU629_RS02985	PTS system, cellobiose-specific IIC component	-2.382700178
QU629_RS20615	QU629_RS20615	Glutaminase	-2.387775421
mglC	QU629_RS04210	Galactose/methyl galactoside ABC transporter	-2.390588789
QU629_RS19205	QU629_RS19205	Sigma-fimbriae tip adhesin	-2.410485582
QU629_RS15345	QU629_RS15345	Outer membrane porin	-2.42559093
QU629_RS09215	QU629_RS09215	Metal-dependent hydrolases of the beta-lactamase superfamily III	-2.429092323
uspG	QU629_RS13740	Universal stress protein G	-2.433261418
adhP	QU629_RS20250	Alcohol dehydrogenase	-2.436018228
ytfT	QU629_RS12325	Galactofuranose ABC transporter	-2.437409319
phoB	QU629_RS17265	Phosphate regulon transcriptional regulatory protein PhoB	-2.464567457
QU629_RS12265	QU629_RS12265	3'(2'),5'-bisphosphate nucleotidase	-2.491076905
QU629_RS08015	QU629_RS08015	Periplasmic alpha-amylase	-2.501565877
QU629_RS10285	QU629_RS10285	Putative inner membrane protein	-2.585834068
aldA	QU629_RS06280	Aldehyde dehydrogenase A	-2.593853124
QU629_RS11740	QU629_RS11740	Type I secretion system, outer membrane component LapE	-2.610692629
QU629_RS09210	QU629_RS09210	Transcriptional regulator	-2.628336257
QU629_RS09225	QU629_RS09225	ABC transporter, substrate-binding protein	-2.644869925
QU629_RS09370	QU629_RS09370	Putative aldolase Z5687	-2.649073561
sstT	QU629_RS13510	Serine/threonine:Na+ symporter SstT	-2.650740268
QU629_RS09365	QU629_RS09365	PfkB family carbohydrate kinase Z5686	-2.655168753
QU629_RS21960	QU629_RS21960	ABC transporter, ATP-binding protein	-2.660521364
QU629_RS18490	QU629_RS18490	Ferric iron ABC transporter, permease protein	-2.711792917
argA	QU629_RS15105	N-acetylglutamate synthase	-2.713661422
csiE	QU629_RS10610	Stationary phase inducible protein CsiE	-2.718686595
acs	QU629_RS09290	Acetyl-CoA synthetase	-2.772951223
QU629_RS06260	QU629_RS06260	RcnR-like protein clustered with cobalt-zinc-cadmium resistance protein CzcD	-2.779781485
QU629_RS19200	QU629_RS19200	Sigma-fimbriae usher protein	-2.81370185
QU629_RS20745	QU629_RS20745	ABC transporter, substrate-binding protein	-2.81932441
ugpB	QU629_RS09915	Glycerol-3-phosphate ABC transporter	-2.842658717
malT	QU629_RS09825	Transcriptional activator of maltose regulon	-2.860552946
potB	QU629_RS01450	Spermidine/putrescine import ABC transporter permease protein PotB	-2.867483145
QU629_RS19130	QU629_RS19130	L-arabinose ABC transporter, ATP-binding protein AraG	-2.872784579

pstS	QU629_RS08720	Phosphate ABC transporter, substrate-binding protein PstS	-2.882402586
QU629_RS06225	QU629_RS06225	Putative insecticidal toxin complex	-2.9022465
pckA	QU629_RS09755	Phosphoenolpyruvate carboxykinase	-2.905022396
QU629_RS08560	QU629_RS08560	Putative transport protein YidE	-2.921705908
actP	QU629_RS09280	Acetate permease ActP	-2.990762449
QU629_RS04715	QU629_RS04715	TRAP-type C4-dicarboxylate transport system	-3.023100505
QU629_RS12165	QU629_RS12165	Methyl-accepting chemotaxis protein	-3.039949425
QU629_RS09380	QU629_RS09380	ABC transport system	-3.097567085
QU629_RS10335	QU629_RS10335	Na+/H+-dicarboxylate symporter	-3.112625944
ytfR	QU629_RS12320	Galactofuranose ABC transporter, permease	-3.120897644
phoE	QU629_RS17620	Outer membrane porin PhoE	-3.148966728
QU629_RS19190	QU629_RS19190	Sigma-fimbriae uncharacterized paralogous subunit	-3.160363249
malF	QU629_RS09045	Maltodextrin ABC transporter, permease protein MdxF	-3.179491339
malE	QU629_RS09050	Maltodextrin ABC transporter, substrate-binding protein MdxE	-3.193786666
QU629_RS15670	QU629_RS15670	Predicted transcription regulator, contains HTH domain	-3.208283312
QU629_RS21965	QU629_RS21965	3',5'-cyclic-nucleotide phosphodiesterase	-3.220107924
malK	QU629_RS09055	Maltose/maltodextrin transport ATP-binding protein MalK	-3.242181458
galS	QU629_RS04195	Mgl repressor and galactose	-3.255010962
mglB	QU629_RS04200	Galactose/methyl galactoside ABC transporter,	-3.256658416
mglA	QU629_RS04205	Galactose/methyl galactoside ABC transporter	-3.273495988
phoA	QU629_RS17330	Alkaline phosphatase	-3.310009815
QU629_RS09220	QU629_RS09220	ABC transporter, ATP-binding protein	-3.312693339
QU629_RS19195	QU629_RS19195	Sigma-fimbriae chaperone protein	-3.323961172
QU629_RS09385	QU629_RS09385	ABC transport system, permease protein Z5690	-3.335360399
QU629_RS09390	QU629_RS09390	ABC transport system, ATP-binding protein	-3.369767728
QU629_RS09060	QU629_RS09060	Maltoporin (maltose/maltodextrin high-affinity receptor	-3.415332548
fadE	QU629_RS17675	Acyl-coenzyme A dehydrogenase FadE	-3.449076598
QU629_RS21975	QU629_RS21975	N-Acetyl-D-glucosamine ABC transport system	-3.527940276
fadB	QU629_RS22045	Enoyl-CoA hydratase	-3.583000353
phnH	QU629_RS09410	Alpha-D-ribose 1-methylphosphonate 5-triphosphate synthase subunit PhnH	-3.729976056
ytfQ	QU629_RS12315	Galactofuranose ABC transporter, ATP-binding protein	-3.87645617
QU629_RS00010	QU629_RS00010	Rhodanese-related sulfurtransferase	-3.927718162
QU629_RS04710	QU629_RS04710	4,5-DOPA dioxygenase extradiol	-4.000897945

phnF	QU629_RS09415	Transcriptional regulator PhnF	-4.038987359

Table S5. Top differentially expressed genes in *K. cowanii* Ch1 during interaction with *S. rolfsii* in presence of VOCs.

**Gene**	**ID**	**Description**	**Log2FoldChange**
fes	QU629_RS14015	Enterobactin esterase	3.634468125
QU629_RS02930	QU629_RS02930	Pyruvate formate-lyase activating enzyme	3.397378079
QU629_RS02935	QU629_RS02935	Pyruvate formate-lyase (EC 2.3.1.54)	3.236784358
ibpB	QU629_RS08580	16 kDa heat shock protein B	3.218758972
gatY	QU629_RS13385	Tagatose-1,6-bisphosphate aldolase GatY	2.923371661
garD	QU629_RS13390	D-galactarate dehydratase	2.897074955
fepB	QU629_RS13980	Ferric enterobactin-binding periplasmic protein FepB	2.89548868
flhA	QU629_RS11585	Flagellar biosynthesis protein FlhA	2.84693832
ibpA	QU629_RS08585	16 kDa heat shock protein A	2.79349997
katG	QU629_RS20195	Catalase-peroxidase KatG	2.736845082
QU629_RS02135	QU629_RS02135	3-hydroxyacyl-[acyl-carrier-protein] dehydratase	2.580207016
QU629_RS13925	QU629_RS13925	Hydroxycarboxylate dehydrogenase (NADP+)	2.453291239
efeU	QU629_RS01960	Ferrous iron transport permease EfeU	2.372193453
QU629_RS13915	QU629_RS13915	5-methylthioribose ABC transporter, permease protein	2.329018541
QU629_RS02605	QU629_RS02605	Virulence factor VirK	2.288018756
fabF	QU629_RS20110	3-oxoacyl-[acyl-carrier-protein] synthase, KASII	2.282064347
QU629_RS02130	QU629_RS02130	Acyl carrier protein	2.262322307
nac	QU629_RS14595	Nitrogen assimilation regulatory protein Nac	2.246135855
gatZ	QU629_RS13380	Tagatose-6-phosphate kinase GatZ	2.222163364
QU629_RS13910	QU629_RS13910	5-methylthioribose ABC transporter, substrate-binding protein	2.218596734
QU629_RS16935	QU629_RS16935	Methionine ABC transporter substrate-binding protein	2.20602317
QU629_RS21570	QU629_RS21570	Antibiotic biosynthesis monooxygenase	2.175185941
flgE	QU629_RS11480	Flagellar hook protein	2.120121576

QU629_RS10705	QU629_RS10705	PTS system, maltose and glucose-specific IIC component	2.09028697
QU629_RS16940	QU629_RS16940	Methionine ABC transporter ATP-binding protein	2.089074811
QU629_RS14020	QU629_RS14020	TonB-dependent receptor; Outer membrane receptor for ferric enterobactin and colicins B, D	2.048495592
QU629_RS11420	QU629_RS11420	ABC transporter, ATP-binding protein	2.039953724
trxC	QU629_RS10450	Thioredoxin 2	2.006859214
QU629_RS13920	QU629_RS13920	5-methylthioribose ABC transporter	1.909576163
mlaA	QU629_RS03525	Outer-membrane-phospholipid-binding lipoprotein MlaA	1.906214479
queG	QU629_RS12045	Epoxyqueuosine reductase	1.848745649
fhuF	QU629_RS20585	Ferric reductase	1.838637761
QU629_RS11415	QU629_RS11415	Ferric iron ABC transporter	1.833960795
flgH	QU629_RS11495	Flagellar L-ring protein FlgH	1.83014165
QU629_RS20365	QU629_RS20365	Iron compound ABC transporter, permease protein	1.817662103
QU629_RS16930	QU629_RS16930	2-Oxobutyrate oxidase, putative	1.790803044
entS	QU629_RS13985	Enterobactin exporter EntS	1.780319284
QU629_RS10700	QU629_RS10700	Maltose-6'-phosphate glucosidase	1.752457258
QU629_RS05045	QU629_RS05045	Polyketide synthase modules and related proteins	1.751705058
flgC	QU629_RS11475	Flagellar basal-body rod modification protein	1.744778872
QU629_RS21790	QU629_RS21790	Zinc ABC transporter	1.740741877
QU629_RS21795	QU629_RS21795	Zinc ABC transporter	1.718010506
efeO	QU629_RS01955	Ferrous iron transport periplasmic protein EfeO	1.657991142
QU629_RS13885	QU629_RS13885	ABC transporter	1.585032688
QU629_RS02125	QU629_RS02125	Putative aminomethyltransferase	1.569963045
QU629_RS13895	QU629_RS13895	S-methyl-5-thioribose-1-phosphate isomerase	1.562646139
gntX	QU629_RS09800	Competence protein F homolog	1.557357797
bfd	QU629_RS21555	Bacterioferritin-associated ferredoxin	1.551490499
QU629_RS20385	QU629_RS20385	Ferrichrome-iron receptor	1.548242669
exbB	QU629_RS04865	TonB-ExbBD energy transducing system	1.547372024
fepD	QU629_RS13995	Ferric enterobactin transport system permease protein FepD	1.509803716
QU629_RS11530	QU629_RS11530	Flagellar transcriptional activator FlhD	1.509171296
fhuA	QU629_RS07035	Ferric hydroxamate outer membrane receptor FhuA	1.502604411
mgrB	QU629_RS16380	PhoP/PhoQ regulator MgrB	1.498074925
cysD	QU629_RS15175	Sulfate adenylyltransferase subunit 2	1.496869378
modA	QU629_RS19485	Molybdenum ABC transporter	1.47394618
QU629_RS20360	QU629_RS20360	Iron compound ABC transporter	1.472482678
znuC	QU629_RS16225	Zinc ABC transporter	1.446075637
QU629_RS16740	QU629_RS16740	Uncharacterized metabolite ABC transporter in Enterobacteriaceae	1.441358506
QU629_RS16945	QU629_RS16945	Methionine ABC transporter permease protein	1.440116137
QU629_RS03965	QU629_RS03965	Phosphotransferase RcsD	1.399293946
QU629_RS20030	QU629_RS20030	L-serine dehydratase, beta subunit	1.37786936
hemH	QU629_RS16830	Ferrochelatase, protoheme ferro-lyase	1.375733027
QU629_RS02335	QU629_RS02335	Alkanesulfonate ABC transporter substrate-binding protein SsuA	1.369885256

nrdI	QU629_RS11315	Ribonucleotide reduction protein	1.356769139
traT	QU629_RS15625	IncF plasmid conjugative transfer surface exclusion protein TraT	1.332364041
zinT	QU629_RS17465	Metal-binding protein	1.322624585
exbD	QU629_RS04870	TonB-ExbBD energy transducing system	1.317687019
QU629_RS02940	QU629_RS02940	Sugar phosphatase YbiV	1.311707229
QU629_RS17860	QU629_RS17860	Glycerol kinase	1.289446287
xylF	QU629_RS07990	D-xylose ABC transporter	1.289272428
modB	QU629_RS19480	Molybdenum ABC transporter permease protein ModB	1.274149005
feoA	QU629_RS09770	Ferrous iron transporter-associated protein	1.271800129
QU629_RS05185	QU629_RS05185	Twitching motility protein	1.2699509
QU629_RS21800	QU629_RS21800	Nicotinamide-nucleotide amidase	1.2627532
cysN	QU629_RS15180	Sulfate adenylyltransferase subunit 1	1.256047609
QU629_RS07995	QU629_RS07995	D-xylose ABC transporter	1.215545036
thiG	QU629_RS21260	Thiazole synthase	1.213475875
QU629_RS19045	QU629_RS19045	BarA-associated response regulator UvrY	1.137501261
QU629_RS06505	QU629_RS06505	Enoyl-[acyl-carrier-protein] reductase	1.128844919
traT	QU629_RS07165	IncF plasmid conjugative transfer surface exclusion protein TraT	1.043258368
QU629_RS04920	QU629_RS04920	Glutathione S-transferase	-1.137461364
lpxP	QU629_RS00620	Lipid A biosynthesis palmitoleoyltransferase	-1.141768336
QU629_RS05825	QU629_RS05825	Tellurium resistance protein TerD	-1.15765179
puuE	QU629_RS20045	Uricase (urate oxidase)	-1.218831477
hutX	QU629_RS17455	Putative heme iron utilization protein	-1.236500297
QU629_RS22035	QU629_RS22035	3-ketoacyl-CoA thiolase	-1.258519591
QU629_RS11755	QU629_RS11755	tmRNA-binding protein SmpB	-1.313288727
QU629_RS20050	QU629_RS20050	Transcriptional regulator, GntR family	-1.336443374
QU629_RS05925	QU629_RS05925	Nitrate ABC transporter, substrate-binding protein	-1.391599029
QU629_RS15670	QU629_RS15670	Predicted transcription regulator	-1.401593831
QU629_RS07915	QU629_RS07915	Antitoxin to RelE-like translational repressor toxin	-1.474898771
QU629_RS20640	QU629_RS20640	Altronate oxidoreductase	-1.488505669
QU629_RS20225	QU629_RS20225	Stress response diiron-containing protein YciF	-1.603197583
amtB	QU629_RS17080	Ammonium transporter	-1.610427948
oxlT	QU629_RS08565	Uncharacterized MFS-type transporter	-1.751503584
QU629_RS17460	QU629_RS17460	Metal-binding protein ZinT	-1.977493301
nac	QU629_RS14600	Alkanesulfonate utilization operon LysR-family regulator Cbl	-2.222223145
QU629_RS06260	QU629_RS06260	RcnR-like protein clustered with cobalt-zinc-cadmium resistance protein CzcD	-2.510240798

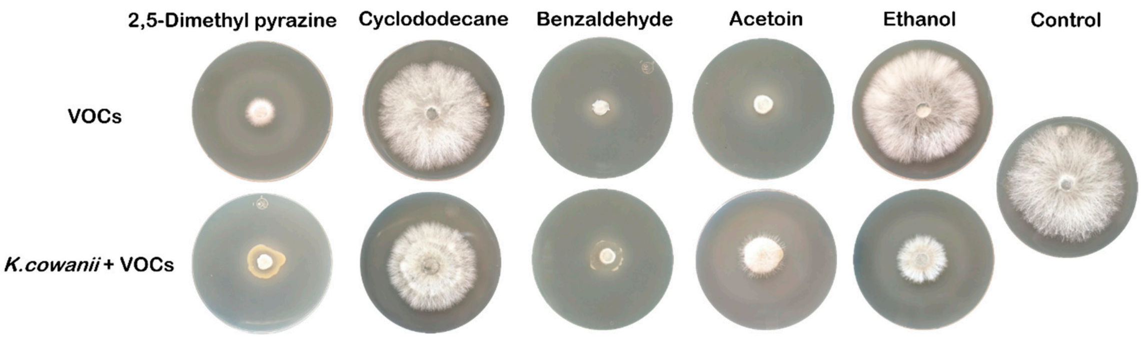

Figure S1. Evaluation of synthetic VOCs on interaction of *K. cowanii* Ch1 and *S. rolfsii* using the double-compartment Petri dish chamber. The treatments applied were: 2,5-Dimethyl pyrazine (50 µL), cyclododecane (50 µL of a solution prepared with 20 mg dissolved in 1 mL of hexane), benzaldehyde (20 µL), acetoin (100 µL) and ethanol (200 µL). CT = control without treatment.

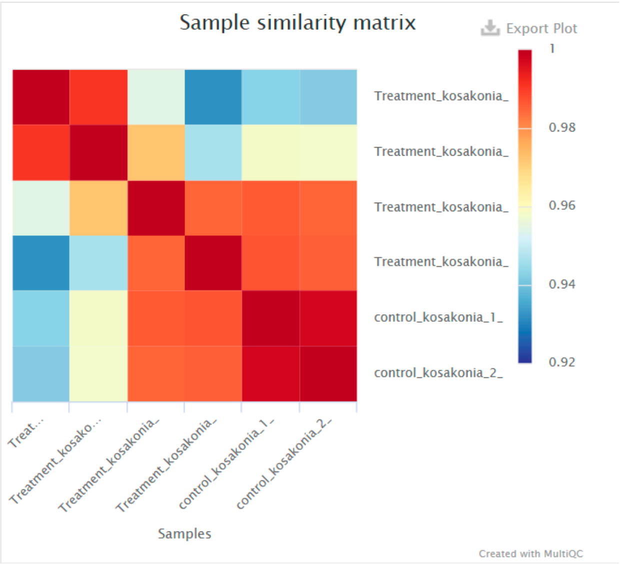

Figure S2. Similarities between samples. Larger values indicate higher similarity between samples. The similarities were calculated using normalized and 'rlog' transformed read counts of all genes using DESeq2.

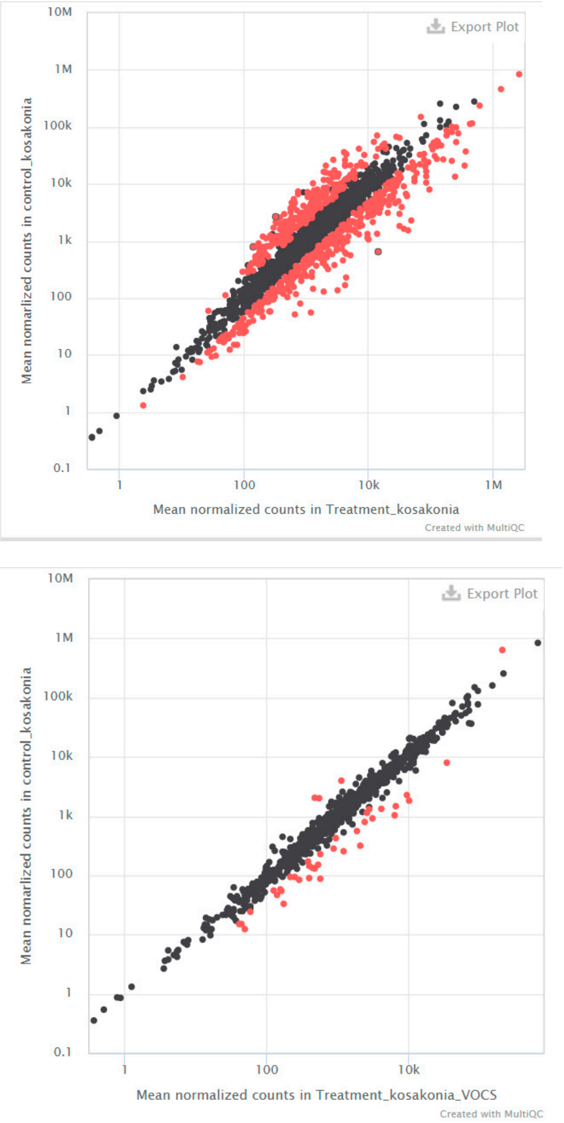

Figure S3. Scatter plot to visualize differential gene expression results. Mean transformed read counts of genes in one group are shown on X-axis while those in the other are shown on Y-axis. Red dots represent differentially expressed genes (adjusted p-values<0.05). Grey dots represent non-differentially expressed genes. Count data transformation was carried out using the 'rlog' method in DESeq2.
